# BNIP3 induction by hypoxia stimulates FASN-dependent free fatty acid production enhancing therapeutic potential of umbilical cord blood-derived human mesenchymal stem cells

**DOI:** 10.1016/j.redox.2017.07.004

**Published:** 2017-07-04

**Authors:** Hyun Jik Lee, Young Hyun Jung, Gee Euhn Choi, So Hee Ko, Sei-Jung Lee, Sang Hun Lee, Ho Jae Han

**Affiliations:** a Department of Veterinary Physiology, College of Veterinary Medicine, Research Institute for Veterinary Science, and BK21 PLUS Program for Creative Veterinary Science Research Center, Seoul National University, Seoul 08826, Republic of Korea; b Department of Pharmaceutical Engineering, Daegu Haany University, Gyeongsan 38610, Republic of Korea; c Medical Science Research Institute, Soonchunhyang University Seoul Hospital, Seoul, Republic of Korea; d Departments of Biochemistry, Soonchunhyang University College of Medicine, Cheonan 330-930, Republic of Korea

**Keywords:** Annexin V, FITC-conjugated annexinV, BCA, bicinchoninic acid, BNIP3, Bcl2/adenovirus E1B 19 kDa protein-interacting protein 3, CBP, CREB-binding protein, CCCP, carbonyl cyanide m-chlorophenyl hydrazine, CHIP, chromatin immunoprecipitation, CHOP, CCAAT-enhancer binding protein homologous protein, CM-H2DCFDA, 2′,7′-dichlorodihydrofluorescein diacetate, CPT1A, carnitine palmitoyltransferase 1A, DGAT1, diglyceride acyltransferase 1, eIF-2α, eukaryotic initiation factor 2 alpha, FA, fatty acid, FASN, fatty acid synthase, FFA, Free fatty acid, FITC, fluorescein isothiocyanate, FOXO3, forkhead box, class O3, FUNDC1, FUN14 domain containing 1, GPAT1, glycerol-3-phsphate acyltransferase 1, HIF-1α, hypoxia-inducible factor-1 alpha, HMSCs, human mesenchymal stem cells, HNA, human nuclear antigen, MAGL, monoacylglycerol lipase, mtROS, mitochondrial ROS, NAC, *N*-acetyl-L-cysteine, NT, non-targeting, PA, palmitic acid, PBA, 4-phenyl butyric acid, PBS, phosphate buffered solution, PI, propidium iodide, PINK1, PTEN-induced putative kinase1, ROS, reactive oxygen species, S.E.M, standard error of mean, SCD1, stearoyl-CoA desaturase 1, SREBP1, sterol regulatory element binding protein 1, TBST, tris-buffered saline containing 0.1% Tween-20, TMRE, tetramethylrhodamine ethyl ester, UCB-hMSCs, umbilical cord blood-derived hMSCs, Hypoxia, Bcl2/adenovirus E1B 19 kDa protein-interacting protein 3 (BNIP3), Mitophagy, Fatty acid synthase (FASN), Mesenchymal stem cell

## Abstract

Mitophagy under hypoxia is an important factor for maintaining and regulating stem cell functions. We previously demonstrated that fatty acid synthase (FASN) induced by hypoxia is a critical lipid metabolic factor determining the therapeutic efficacy of umbilical cord blood-derived human mesenchymal stem cells (UCB-hMSCs). Therefore, we investigated the mechanism of a major mitophagy regulator controlling lipid metabolism and therapeutic potential of UCB-hMSCs. This study revealed that Bcl2/adenovirus E1B 19 kDa protein-interacting protein 3 (BNIP3)-dependent mitophagy is important for reducing mitochondrial reactive oxygen species accumulation, anti-apoptosis, and migration under hypoxia. And, BNIP3 expression was regulated by CREB binding protein-mediated transcriptional actions of HIF-1α and FOXO3. Silencing of BNIP3 suppressed free fatty acid (FFA) synthesis regulated by SREBP1/FASN pathway, which is involved in UCB-hMSC apoptosis via caspases cleavage and migration via cofilin-1-mediated F-actin reorganization in hypoxia. Moreover, reduced mouse skin wound-healing capacity of UCB-hMSC with hypoxia pretreatment by BNIP3 silencing was recovered by palmitic acid. Collectively, our findings suggest that BNIP3-mediated mitophagy under hypoxia leads to FASN-induced FFA synthesis, which is critical for therapeutic potential of UCB-hMSCs with hypoxia pretreatment.

## Highlights

• BNIP3 induction by hypoxia mainly controls mitophagy and mitochondrial ROS production in UCB-hMSCs.
• BNIP3 silencing impairs UCB-hMSC functions such as survival, migration and free fatty acid production under hypoxia.
• BNIP3 silencing suppresses SREBP1/FASN-mediated free fatty acid production via ROS regulation under hypoxia.
• BNIP3 silencing decreased skin wound healing potential of hypoxia-pretreated UCB-hMSCs.
• Palmitic acid addition recovers decreased therapeutic potential of UCB-hMSCs by BNIP3 silencing.

## Graphical abstract

**Figure:**
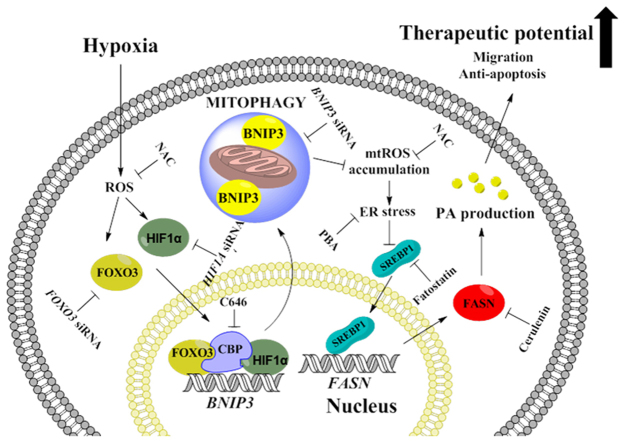

## 1 Introduction

Metabolic alteration of stem cells under hypoxia is prerequisite to controlling stem cell function activated by oxygen signaling [1], [2]. Many investigators have shown that, in regenerative medicine, stem cell regulation with hypoxia has many advantages for stem cell functional regulation compared to that with normoxia [3], [4]. However, the essential metabolic factor that enhances and sustains the functional regulation of stem cells by hypoxia has been incompletely described. Investigation to unveil the relationships between metabolism and stem cell physiology under hypoxia is required to optimize stem cell-based therapy for clinical application in regenerative medicine. As several investigators recently demonstrated, fatty acid (FA) and its metabolites, such as sphigosine-1-phosphate and lysophosphatidic acid produced within the stem cell have the capacity to control the metabolism and bioactivity of stem cells, although it is reported that the role of FA oxidation in ATP synthesis is insignificant [5], [6], [7], [8]. Thus, the interest in the role of lipid metabolism regulation by hypoxia is increasing. Some studies investigating lipid metabolic changes induced by hypoxia reported that hypoxia stimulates FA uptake, de novo FA synthesis, and FA metabolite synthesis [9], [10]. However, there are few studies investigating the role of lipid metabolism altered by hypoxia in stem cell regulation. Despite reactive oxygen species (ROS) accumulation, which causes ischemic injury, the detailed mechanism involved in how stem cells exposed to hypoxia maintain lipid metabolism and function is not fully described. An investigation into factors protecting against impairment of lipid metabolism shift under high ROS accumulation conditions should provide novel insight into the control of stem cells under hypoxia.

Mitophagy is mitochondria-specific autophagy that removes mitochondria in order to maintain mitochondrial quality [11]. It has been shown that mitophagy prevents mitochondrial dysfunction, inflammation, apoptosis, and severe oxidative stress, which are closely associated with pathological progress of neurological and metabolic diseases [12], [13]. Mitophagy is mediated by two mitophagy regulator types, mitophagy receptor and E3 ubiquitin ligase. In addition, some investigators have reported that hypoxia-induced metabolic stress stimulates mitophagy [11], [14]. Mild oxidative stress specifically induces mitophagy without nonspecific autophagy, whereas a high ROS level stimulates both autophagy and mitophagy as a negative-feedback mechanism to reduce mitochondria-derived ROS production [15], [16], [17]. Many researchers have attempted to demonstrate the mechanism for the initial stimulation of mitophagy by oxidative stress. Several mitophagy regulators including mitophagy receptors, such as Bcl2/adenovirus E1B 19 kDa protein-interacting protein 3 (BNIP3), NIX, FUN14 domain containing 1 (FUNDC1), and PTEN-induced putative kinase1 (PINK1)/Parkin, which induce mitophagy in mammalian cells under hypoxia, have been identified [18], [19], [20]. However, there have been no studies investigating the contribution of such mitophagy regulators to mitophagy induced by hypoxia in stem cells. Since there are few studies demonstrating the role of mitophagy in stem cell differentiation, further investigation is required to elucidate the relationships between mitophagy and stem cell regulation under hypoxia [21]. In addition, uncovering the molecular mechanism involved in mitophagy regulation of lipid metabolism in stem cells under hypoxia can answer the question: What is the key player in the induction and maintenance of lipid metabolism in stem cells under hypoxia?

Clinical application of human mesenchymal stem cells (hMSCs) has been considered a promising therapeutic strategy for scarless wound healing and ischemic injury [22], [23]. The hMSC effect is associated with cell replacement and paracrine effects leading to angiogenesis and damaged tissue repair [24], [25], [26]. Umbilical cord blood-derived hMSCs (UCB-hMSCs) are the most abundant non-embryonic cell source and have generated remarkable interest in researchers studying stem cell-based therapy, because of their multiple differentiation potential and immune modulation capacity, as they can be obtained easily and non-invasively without ethical concerns [27], [28]. Therefore, investigation into the regulation of UCB-hMSC physiological function under hypoxia may improve the therapeutic effect of UCB-hMSCs in regenerative medicine. The aim of our investigation is to identify the detailed regulatory mechanism of a major mitophagy regulator controlling lipid metabolism and therapeutic potential of UCB-hMSCs under hypoxia.

## 2 Materials and methods

### 2.1 Materials

The UCB-hMSCs, obtained from Medipost (Seoul, Korea, http://www.medi-post.com). The UCB-hMSCs were positive for HLA-AB but not for HLA-DR, and characterized to express CD73, CD105, but not CD14, CD34, and CD45. Fetal bovine serum (FBS, cat no. SH30088.03IR) and antibiotics (cat no. 15240062) for UCB-hMSC cultivation were purchased from Hyclone (Logan, UT, USA) and Gibco (Grand Island, NY, USA), respectively. Cytochrome c oxidase subunit 4 (COX4, cat no. ab14744), α–smooth muscle actin (α-SMA, cat no. ab5694), and sterol regulatory element binding protein 1 (SREBP1, cat no. ab3259) antibodies were purchased from Abcam (Cambridge, MA, USA). LC3B (cat no. NB100-2220), NIX (cat no. NBP1-88558) and hypoxia inducible factor 1α (HIF-1α, cat no. NB100-105) antibodies were obtained from Novus Biologicals (Littleton, CO, USA). BNIP3 (cat no.sc-56167), β-tubulin (cat no. sc-69966), β-actin (cat no. sc-47778), PINK1 (cat no. sc-33796), Lamin A/C (cat no. sc-20681), CREB-binding protein (CBP, cat no. sc-7300), fatty acid synthase (FASN, cat no. sc-20140), CCAAT-enhancer binding protein homologous protein (CHOP, cat no. sc-793), p-cofilin1 (cat no. sc-12912-R), cofilin1 (cat no.sc-33779) and caspase-9 (cat no.sc-8355) antibodies were purchased from Santa Cruz (Paso Robles, CA, USA). p-S6 (cat no. #2215), p-eukaryotic initiation factor 2α (p-eIF2α, cat no. #9721), eIF2α (cat no. #9722), p-mammalian target of rapamycin (p-mTOR, cat no. #2971), mTOR (cat no. #2983), p-S6K1 (cat no. #9234), cleaved caspase-3 (cat no. #9661) and forkhead box class O3 (FOXO3, cat no. #2497) antibodies were purchased from Cell Signaling Technology (Danvers, MA, USA). Human nuclear antigen (HNA) antibody (cat no. MAB1281) was acquired from EMD Millipore (Billerica, MA, USA). Horseradish peroxidase (HRP)-conjugated rabbit anti-mouse (cat no. PA1-28568) and goat anti-rabbit (cat no. 32460) secondary antibodies were obtained from Thermo Fisher (Waltham, MA, USA). Alexa fluor 488 (cat no. A32731)- and 555(cat no. A21429)-conjugated secondary antibodies and propidium iodide (PI, cat no. P3566) were purchased from Life Technologies (Gaithersburg, MD, USA). CAY10566 (cat no. 10012562) was obtained from Cayman chemical (Ann Arbor, MI, USA). Cerulenin (cat no. C2389), 4-phenyl butyric acid (PBA, cat no. P21005), *N*-acetyl-L-cysteine (NAC, cat no. A7250), C646 (cat no. SML0002) and fatostatin (cat no. F8932) and palmitic acid (PA, cat no. P0500) were acquired from Sigma-Aldrich (St. Louis, MO, USA). Small interfering RNAs (siRNAs) for *PINK1*(cat no. L-004030-00-0005), *BNIP3* (cat no. L-004636-00-0005), *NIX* (cat no. L-011815-00-0005), *FOXO3* (cat no. L-003007-00-0005) and non-targeting (NT, cat no. L-001206-13-20) were purchased from Dharmacon (Lafayette, CO, USA). *HIF1A* siRNA was obtained from Gene Pharma (Gene Pharma, Shanghai, China). All reagents used in the present study were of the highest quality commercially available forms.

### 2.2 Cultivation of UCB-hMSCs

UCB-hMSCs were cultured with α–minimum essential medium (α-MEM; cat no. SH30265.01, Hyclone) containing 10% FBS, 1% antibiotic-antimycotic solution containing penicillin, streptomycin, and fungizone. UCB-hMSCs were plated in 35, 60, or 100 mm diameter culture dishes in an incubator kept at 37 °C with 5% CO_2_. Plated UCB-hMSCs were grown for 4 days and washed with phosphate buffered solution (PBS). Growth medium was changed to serum-free medium prior to pretreatment of reagent or hypoxia.

### 2.3 Hypoxia treatment

A modular hypoxia incubation chamber (Billups-Rothenberg, Del Mar, CA, USA) was used. The hypoxic gas used in this study included 2.2% O_2_, 5% CO_2_ and 92.7% N_2_. The hypoxia incubation chamber was purged with the hypoxic gas at a 5 L/min flow rate for 15 min and then placed in the conventional cell incubator at 37 °C.

### 2.4 Western blot analysis

UCB-hMSCs were washed with ice-cold PBS and harvested with a cell scraper. Collected samples were lysed with RIPA lysis buffer (cat no. 89901, Thermo Fisher) containing proteinase and phosphatase inhibitor (cat no. 78440, Thermo Fisher) for 30 min on ice. The lysates were cleared by centrifugation (13,000×*g*, 4 °C, 30 min). Determination of protein concentration was performed by using a bicinchoninic acid (BCA) detection kit (cat no. 23227, Thermo Fisher). Protein sample (10 µg) was loaded in 8–12% SDS-polyacrylamide gel and transferred to a polyvinylidene fluoride (PVDF) membrane. The protein sample-transferred membrane was washed with tris-buffered saline containing 0.1% Tween-20 (TBST) solution {150 mM NaCl, 10 mM Trish-HCl (pH7.6), 0.1% Tween-20} for 30 min. The membrane was blocked with 5% skim milk (Gibco) or 5% bovine serum albumin (BSA) for 1 h at 4 °C. The membrane was washed with TBST for 30 min three times. Next, the membrane was incubated with primary antibody solution (1:1000 dilution) overnight at 4 °C. Subsequently, it was incubated with secondary antibody solution (1:10,000 dilution) at 4 °C for 4 h. Protein bands were detected by using a chemiluminescence detection kit (cat no. K-12045-D50, Advansta Inc., Menlo Park, CA, USA). Densitometric analysis for quantification of protein bands was performed by using ImageJ software (developed by Wayne Rasband, National Institutes of Health, Bethesda, MD, USA; http://rsb.info.nih.gov.kr/ij/). Full-length gel images are presented in the Supplemental Materials (Sup. Figs. S6-S9).

### 2.5 Preparation of mitochondrial fraction sample

Mitochondrial isolation was performed by using a commercial mitochondrial isolation kit (cat no. 89874, Thermo Fisher) according to the manufacturer's manual. Briefly, harvested samples were incubated in Reagent A for 2 min on ice. Subsequently, the cell lysate sample was incubated with Reagent B for 5 min. Next, Reagent C was added to the lysate sample. The cell lysate sample was centrifugated at 3,000×*g* for 15 min. Supernatant was used as a cytosolic fraction. The pellet was lysed with 2% CHAPS in Tris-buffered saline (25 mM Tris, 0.1 M NaCl, pH 7.2) solution and used as a mitochondrial fraction for 30 min on ice.

### 2.6 Preparation of nuclear fraction sample

Collected samples were suspended with nuclear fractionation buffer solution {137 mM NaCl, 8.1 mM Na_2_HPO_4_, 2.7 mM KCl, 1.5 mM KH_2_PO_4_, 2.5 mM EDTA, 1 mM dithiothreitol, 0.1 mM PMSF, and 10 mg/mL leupeptin (pH 7.5)}. Samples were lysed mechanically with a 23-gauge needle and incubated for 10 min on ice. Cell lysates were centrifugated at 800×*g* for 5 min. Pellet sample, as a nuclear fraction, was washed with PBS and lysed with RIPA lysis buffer for 30 min on ice.

### 2.7 Transfection of siRNA

Prior to treatment of reagent or hypoxia, 20 nM of siRNAs specific for *PINK1*, *BNIP3*, *NIX*, *FOXO3* and NT with transfection reagent TurboFect™ (cat no. R0531, Thermo Fisher) were added to UCB-hMSCs, which were then incubated for 24 h in a conventional cell incubator at 37 °C in 5% CO_2_. The siRNAs sequences used in this study are described in Supplementary Table S3.

### 2.8 Co-immunoprecipitation

To confirm the formation of a protein complex in a cell lysate sample, we performed co-immunoprecipitation with a commercial co-immunoprecipitation kit (cat no. 26149, Thermo Fisher) according to manufacturer's manual. Harvested cells were lysed with IP lysis buffer and incubated for 5 min on ice. Cell debris was cleared by centrifugation at 13,000×*g*, 4 °C for 10 min. Supernatant sample was collected. Determination of protein concentration of lysate sample was performed with a BCA quantification assay (Thermo Fisher). CBP antibody was immobilized with AminoLink Plus coupling resin (Thermo Fisher) for 2 h. Immobilized resin was washed with a commercial wash buffer and incubated with cell lysate for 6 h at 4 °C. IgG antibody was provided in an IP kit and used as a negative control to assess the degree of non-specific binding to resin. Protein samples were analyzed by western blot analysis.

### 2.9 Reverse transcription-polymerase chain reaction (PCR) and real-time PCR

RNA was extracted by using a MiniBEST Universal RNA extraction kit (cat no. 9767, TaKaRa, Otsu, Shiga, Japan). RNA (1 µg) was reverse-transcribed with a Maxime RT-PCR PreMix kit (cat no. 25081, iNtRON Biotechnology, Seongnam, Korea) to produce the cDNA sample. Reverse transcription was performed for 1 h at 45 °C and 5 min at 95 °C. The cDNA sample was amplified with a QuantiNova SYBR kit (cat no. 208054, Life Technologies) and mRNA primers for *FASN*, *SCD1*, *SCD5*, *GPAT1*, *GPAT3*, *GPAT4*, *MAGL*, *DGAT1*, *CPT1A*, and *ACTB*. The expression of *ACTB* mRNA was used for normalization of gene expressions. The primer sequences are described in Supplementary Table S2. Quantitative analysis of mRNA expression was carried out by using a Rotor-Gene 6000 real-time thermal cycling system (Corbett Research, Mortlake, NSW, Australia). Real-time PCR was performed as follows: 10 min at 95 °C for DNA polymerase activation and 50 cycles of 15 s at 94 °C, 20 s at 55 °C, and 30 s at 72 °C. The identity and specificity of the PCR product was validated by performing melting curve analysis.

### 2.10 Measurement of cellular free fatty acid (FFA) production

Cellular FFA was measured by using an FFA quantification colorimetric/fluorometric kit (cat no. K612, Biovision, Mountain View, CA, USA) according to manufacturer's indication. Same numbers of UCB-hMSC samples were collected and incubated with acetyl-CoA synthetase reagent, enhancer solution, and enzyme mixture as provided in the kit. Lipid samples were incubated at 37 °C for 30 min. Cellular FFA levels were measured by using a microplate reader at 550 nm (Bio-Rad).

### 2.11 Chromatin immunoprecipitation (CHIP)

CHIP assay was performed by using EZ-CHIP-Chromatin immunoprecipitation kit (cat no. 17-371RF, EMD Millipore, Billerica, MA, USA) according to the manufacturer's manual. Briefly, samples lysed by sodium dodecyl sulfate (SDS) lysis buffer were incubated with HIF-1α, FOXO3, normal IgG, and Pol III-specific antibodies overnight at 4 °C. Normal IgG and Pol III-specific antibodies were used as negative and positive controls, respectively. Immunoprecipitated protein-chromatin complex samples were eluted with elution buffer provided with the kit {1% SDS, 50 mM Tris-HCl (pH 7.5), 10 mM EDTA}. Eluted samples were incubated with 5 M NaCl at 65 °C for 4 h and subsequently incubated with RNase A at 37 °C for 30 min. Eluates were incubated with 0.5 M EDTA, 1 M Tris-HCl, and proteinase K at 45 °C for 2 h. DNA was acquired by using DNA purification column and amplified by real-time PCR with primer. The primer sequences and target consensus sequences for CHIP assay are described in Supplementary Table S4 and Supplementary Fig. S5.

### 2.12 Immunocytochemistry

UCB-hMSCs were cultured on a confocal dish (cat no. SPL200350, Pocheon, Korea). After reagent and hypoxia treatment, cells were washed with PBS twice and incubated in 80% acetone in PBS for 10 min. Fixed cells were blocked with 5% FBS in PBS and incubated with primary antibody (1:100 dilution) for 2 h at 4 °C, followed by Alexa 488- or 555-conjugated secondary antibody (1:100 dilution) and PI for 1 h at room temperature. Fluorescence images were acquired by confocal microscopy (FluoView™ 300 confocal microscope; Olympus, Tokyo, Japan). Fluorescence intensity within the PI region was analyzed by using ImageJ software.

### 2.13 Trypan blue exclusion cell viability assay

UCB-hMSCs-conditioned medium was harvested, and UCB-hMSCs were washed with PBS and detached with 0.05% trypsin solution (Gibco) and 0.5 mM EDTA. Soybean trypsin inhibitor was added to cell suspension solution for quenching. Suspended cells were added to the collected medium along with the cell suspension solution. The cell suspension solution was centrifugated at 3,000×*g* for 5 min at 4 °C. The obtained pellet was incubated in 0.4% trypan blue (Sigma-Aldrich) solution in PBS to stain the dead cells. Both stained and unstained cells were counted by using a Petroff-Hausser counting chamber (Hausser Scientific, Horsham, PA, USA). To determine cell viability, the following formula was used: Cell viability = [{1– (trypan blue-stained cell number/total cell number)} × 100].

### 2.14 AnnexinV/PI apoptosis detection

To evaluate apoptosis of UCB-hMSCs, fluorescein isothiocyanate-conjugated annexinV (annexinV) and PI-double staining analysis was performed by using an annexinV-FITC apoptosis detection kit (cat no. 556547, BD Bioscience, Franklin Lakes, NJ, USA) according to the supplier's instructions. After treatment, UCB-hMSCs were collected and counted by using a Petroff-Hausser cell counting chamber. Cells (1 × 10^5^) were suspended in binding buffer supplied by a commercial kit. AnnexinV-FITC (5 μL) and PI (5 μL) were added to the cell suspension solution, which was then incubated for 15 min at room temperature. UCB-hMSC apoptosis was measured by using flow cytometry (Beckman Coulter, Fullerton, CA, USA). Cells (3 × 10^3^) that had similar side scatter and forward scatter levels were measured by using flowing software2 (developed by Perttu Terho, Turku, Finland). AnnexinV-negative and PI-negative (Q3) cells were considered viable. AnnexinV-negative and PI-positive (Q1), annexinV-positive and PI-positive (Q2), and annexinV-positive and PI-negative (Q4) were considered as late apoptotic/necrotic, apoptotic and early apoptotic cells, respectively. To determine the percentage of total apoptotic cells, the following formula was used: Apoptotic cells = Q1 + Q2 + Q4.

### 2.15 Measurement of intracellular ROS production

UCB-hMSCs were detached and then counted by using a Petroff-Hausser counting chamber. Cells (1 × 10^6^) were incubated in PBS solution containing 10 μM of CM-H_2_DCFDA (cat no. C6821, Thermo Fisher) at 37 °C and 5% CO_2_ for 30 min. Cells were washed with PBS twice, then loaded into a 96-well plate. Fluorescence intensity of intracellular ROS was assessed by using a plate reader (Victor3; Perkin-Elmer, Waltham, MA, USA) at excitation and emission wavelengths of 485 and 535 nm, respectively.

### 2.16 Measurement of mitochondrial ROS production

To measure the mitochondrial ROS production and mitochondrial membrane potential, a MitoSOX™ (cat no. M36008, Life Technologies) staining kit was used. After treatment, UCB-hMSCs were incubated in 5 μM of MitoSOX™ for 10 min in a conventional cell incubator kept at 37 °C and with 5% CO_2_. Cells were washed with PBS twice and detached with 0.05% trypsin solution. Collected cells were washed and analyzed by using a flow cytometer (Beckman Coulter). Cells (3 × 10^3^) that had similar side scatter and forward scatter levels were measured by using Flowing Software 2 (developed by Perttu Terho, Turku, Finland).

### 2.17 Measurement of mitochondrial volume and mitochondrial membrane potential

To measure the mitochondrial volume and membrane potential, a Mitotracker™ (cat no. M7514, Life Technologies) staining kit and TMRE (cat no. 87917, Sigma-Aldrich) were used, respectively. After treatment, UCB-hMSCs were incubated in 200 nM of Mitotracker™ or 200 nM of tetramethylrhodamine, ethyl ester (TMRE) for 15 min at room temperature. Live UCB-hMSCs were washed with PBS solution twice and detached by using 0.05% trypsin solution. Subsequently, cells were washed with PBS twice. Fluorescence intensities of Mitotracker™ or TMRE were detected by using flow cytometry. Cells (5 × 10^3^) that had similar side scatter and forward scatter levels were measured by using Flowing Software 2.

### 2.18 ibidi™ insert dish migration assay

Cells (1 × 10^4^) were plated onto each well of an ibidi™ insert dish (cat no. 81176, ibidi, Martinsried, Germany) which has two reservoirs separated by a 500 μm-thick silicon wall. Cells were grown in the complete medium until cells reached full confluence. Next, reagents were pretreated to the cells and incubated under hypoxia for 24 h as hypoxia pretreatment. After incubation, reservoirs were gently eliminated and cells incubated under normoxia for 12 h. Cells were immunostained with FITC-conjugated phalloidin (cat no. A12379, Thermo Fisher) and PI. Cells were fixed with 80% acetone, blocked in 5% FBS in PBS solution. After washing with PBS twice, cells were incubated with FITC-conjugated phalloidin and PI for 1 h at room temperature. Fluorescence images were acquired by using a confocal microscope.

### 2.19 Oris™ migration assay

Cells (1 × 10^4^) were plated onto each well of an Oris™ migration assay plate (cat no. CMACC1.101, Platypus Technologies, Fitchburg, WI, USA). For hypoxia pretreatment, plates were incubated under hypoxia for 24 h. Insert plugs were gently removed, and plates were incubated under normoxia for 24 h. Cells were washed with PBS twice and incubated with 5 μΜ of calcein AM (cat no. C1430, Thermo Fisher) for 30 min. Cells that migrated into the denuded well area were detected by using a microplate reader (Perkin-Elmer) at excitation and emission wavelengths of 485 nm and 535 nm, respectively.

### 2.20 Mouse skin wound healing model

All procedures involving animal were conducted following the National Institutes of Health Guidelines for Humane Treatment of Animals and with approval from the Institutional Animal Care and Use Committee of Seoul National University (SNU-140123-6). Eight-week-old male Institute for Cancer Research (ICR) mice were used. All mice were anesthetized with a 2:1:2 mixture of Zoletil™ (20 mg/kg; Virbac Laboratories, Carros, France), xylazine HCl (10 mg/kg; Rompun™, Bayer, Leverkusen, Germany), and normal saline prior to skin wound surgery. All surgery was performed by three authors who have a doctor of veterinary medicine license granted by the Ministry of Agriculture and Forestry of Republic of Korea. Every effort was made to minimize suffering during skin wound surgery. Mouse skin wound healing with stem cell transplantation was performed as previously described [29]. The back of the anesthetized mouse was shaved, scrubbed with an organic iodine solution and 70% ethanol solution for disinfection. A 6 mm diameter circular wound was surgically created by sterile biopsy punch. BNIP3 siRNA or NT siRNA-transfected UCB-hMSCs were pretreated with hypoxia or PA for 24 h. Experimental mice were divided into six groups: mice given vehicle (group 1, *n* = 6); mice given NT siRNA-transfected UCB-hMSCs (group 2, *n* = 6); mice given NT siRNA-transfected UCB-hMSCs with hypoxia pretreatment (group 3, *n* = 6); mice given *BNIP3* siRNA transfected UCB-hMSCs with hypoxia pretreatment (group 4, *n* = 6); mice given *BNIP3* siRNA-transfected UCB-hMSCs with hypoxia and PA pretreatment (group 5, *n* = 6); and mice given *BNIP3* siRNA-transfected UCB-hMSCs (group 6, *n* = 6). Cells (1 × 10^6^) in 100 μL of PBS were injected to the dermis intradermally at three sites around each circular wound. All wound images were acquired at the same distance from the object (30 cm) with a digital camera system (D50; Nikon, Tokyo, Japan) at post-injection days 0, 4, 8 and 12. All wounds were covered with Tegaderm™ (3M, London, Canada). Measurement of wound area was determined by using ImageJ software. Wound closure was measured as the difference in wound area on a particular day compared to day 0. At post-injection day 12, all mice were euthanized. The skin wound samples were embedded in O.C.T. compound (cat no. 4583, Sakura Finetek, CA, USA), and stored at −80 °C. Frozen samples were sectioned to a 10 µm thickness by using a cryostat (Leica CM 1520, Leica, Wetzlar, Germany) and then mounted on SuperFrost Plus slides (Thermo Fisher) for hematoxylin and eosin (H&E) staining and immunohistochemistry assessment. Vessel intensity measurement was analyzed by using ImageJ software.

### 2.21 Histological examination

Slide samples were fixed with 4% paraformaldehyde (cat no. LGB-1175, Lugen Sci, Seoul, Korea) and stained with H&E for 5 min. Samples were washed with 95% and 100% ethanol three times and incubated in xylene for 5 min. All H&E stained sample images were acquired by using light microscopy. Histological evaluation and re-epithelization scoring were performed in a blind fashion. The scoring of re-epithelization during wound healing with H&E-stained tissue samples was evaluated by determining the percentage of tissue showing the qualitative features of re-epithelization according to a method described previously [30].

### 2.22 Immunohistochemistry

Skin tissue samples on slides were fixed in 80% acetone solution. Subsequently, slide samples were washed and then blocked in 5% normal goat serum (cat no. 566380, Sigma-Aldrich) in PBS for 30 min. Slides were incubated with primary (1:100 dilution) for 2 h. After washing three times with PBS, slides were incubated with Alexa 488-conjugated secondary antibody with PI (1:100 dilution) for 1 h. Slide samples were visualized by using confocal microscopy. All images were analyzed by using MetaMorph software (Universal Imaging, West Chester, PA, USA).

### 2.23 Statistical analysis

All data shown in the results are presented as a mean ± standard error of mean (S.E.M.). Statistical differences among experimental samples were analyzed by using analysis of variance. Comparisons of treatment groups with control groups were performed by using the Student's test. A *p*-value < 0.05 was considered statistically significant.

## 3 Results

### 3.1 Regulatory effect of BNIP3 on UCB-hMSC mitophagy under hypoxia

**Fig. 1:**
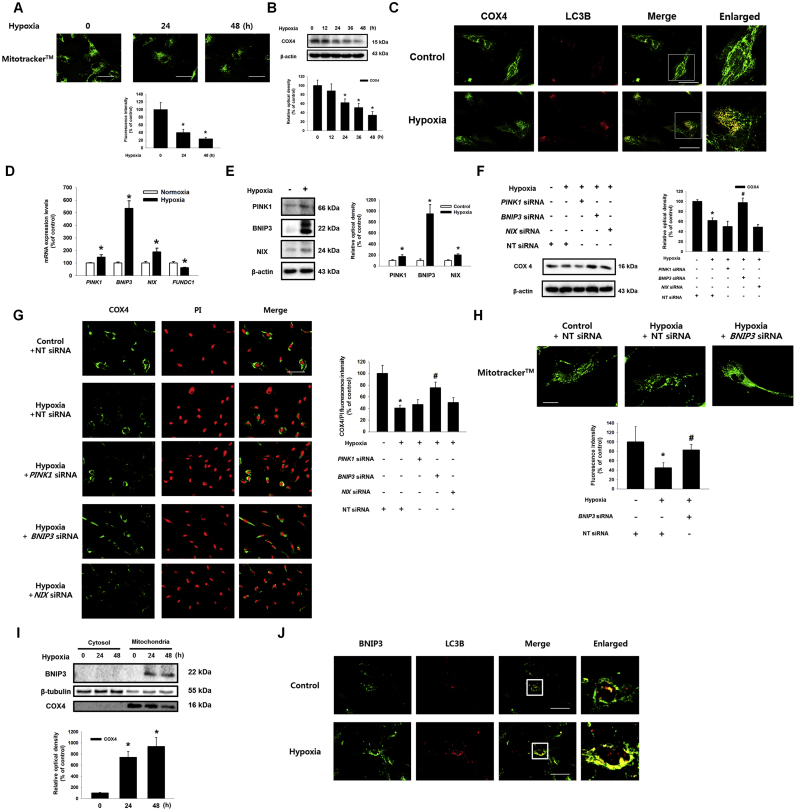
**Effects of hypoxia on mitophagy regulator expressions and mitophagy in UCB-hMSCs. (**A) UCB-hMSCs were incubated with various times of hypoxia (0–48 h). Cells were stained with Mitotracker™. *n* = *6* (magnification, ×1,200). Scale bars, 50 µm. (B) The expressions of COX4 and β-actin were detected by western blot. *n* = *4*. (C) UCB-hMSCs were exposed to 24 h of normoxia or hypoxia. Cells were immuno-stained with COX4 and LC3B-specific antibodies (magnification, ×600). Scale bars, 37.5 µm. (D) The mRNA expressions of *PINK1*, *BNIP3*, *NIX* and *FUNDC1* were analyzed by quantitative real-time PCR (qPCR). *n* = *5.* (E) The protein expressions of PINK1, BNIP3, NIX and β-actin were assessed by western blot. *n* = *4.* (F, G) siRNAs of *PINK1*, *BNIP3*, *NIX* or non-targeting (NT) were transfected to UCB-hMSCs prior to hypoxia treatment for 24 h. COX4 and β-actin expressions were assessed by western blot. *n* = *4* (F). Cells were immunostained with COX4 and PI. *n* = *3* (magnification, ×400). All scale bars, 50 µm. COX4 fluorescence intensity was analyzed by luminometer. *n* = *5* (G). (H) *BNIP3* siRNA was transfected to UCB-hMSCs prior to hypoxia treatment for 24 h. Cells were stained with Mitotracker™. *n* = *6* (magnification, ×1,200). Scale bars, 50 µm. (I) BNIP3, β-tubulin and COX4 with cytosol and mitochondrial fractionized samples were detected by western blot. (J) Cells were incubated with hypoxia or normoxia for 24 h. Cells were immuno-stained with BNIP3 and LC3B-specific antibodies. (magnification, ×600). Scale bars, 37.5 µm. Western blot data were normalized by β-actin, and qPCR data were normalized by *ACTB* mRNA expression level. Quantitative data are presented as a mean ± S.E.M. All blot and confocal images are representative. *p < 0.05 versus control, *#p* < 0.05 versus hypoxia.

To determine the effect of hypoxia on mitophagy in UCB-hMSCs, the cells were incubated under hypoxia for various durations (0–48 h). First, we measured mitochondrial volume in UCB-hMSCs under hypoxia by using a mitochondria specific fluorescent dye, Mitotracker™. The fluorescence intensities of Mitotracker™ in the 24 h and 48 h hypoxia-treated UCB-hMSCs decreased to 40.2% and 23.6%, respectively, of the control level (Fig. 1A). COX4 protein expression decreased in a time-dependent manner during the 24–48 h of hypoxia (Fig. 1B). Immunofluorescence results showed that 24 h of hypoxia stimulated co-localization of COX4 with LC3B (Fig. 1C). In addition, we observed that ATP production in hypoxia-treated cells decreased to 73.2% of the control level (Sup. Fig. S1). We checked the mRNA expressions of mitophagy regulator genes, such as *PINK1*, *BNIP3*, *NIX* and *FUNDC1*, to determine the effect of hypoxia on expression of mitophagy regulators in UCB-hMSCs. As shown in the Fig. 1D, *PINK1*, *BNIP3* and *NIX* mRNA expressions in UCB-hMSC significantly were increased, whereas *FUNDC1* mRNA expression was reduced by hypoxia. PCR and western blotting results showed that, among the tested genes, *BNIP3* expression was increased to the greatest extent by hypoxia (Fig. 1D and E). We transfected *PINK1*, *BNIP3*, *NIX* and non-targeting (NT) siRNAs, and assessed COX4 expression in UCB-hMSCs to confirm the effect of mitophagy regulators induced by hypoxia on mitophagy in UCB-hMSCs under hypoxia. We also confirmed the transfection of siRNAs of *PINK1*, *BNIP3* and *NIX* significantly decreased their mRNA expressions, respectively (Sup. Fig. S2). The western blot and immunofluorescence staining results showed the decrease in COX4 expression by hypoxia was significantly recovered by *BNIP3* siRNA transfection (Fig. 1F and G). The fluorescent intensity of Mitotracker™ in the *BNIP3* siRNA-transfected UCB-hMSCs under hypoxia was higher than that in NT siRNA-transfected UCB-hMSCs under hypoxia (Fig. 1H). In addition, hypoxia increased BNIP3 expression in the mitochondrial fraction and co-localization of BNIP3 with LC3B (Fig. 1I and J). Collectively, our results suggest that BNIP3 up-regulation by hypoxia mainly induces mitophagy via LC3B in UCB-hMSCs.

### 3.2 Role of BNIP3 in the regulation of ROS accumulation and functions in UCB-hMSCs under hypoxia

**Fig. 2:**
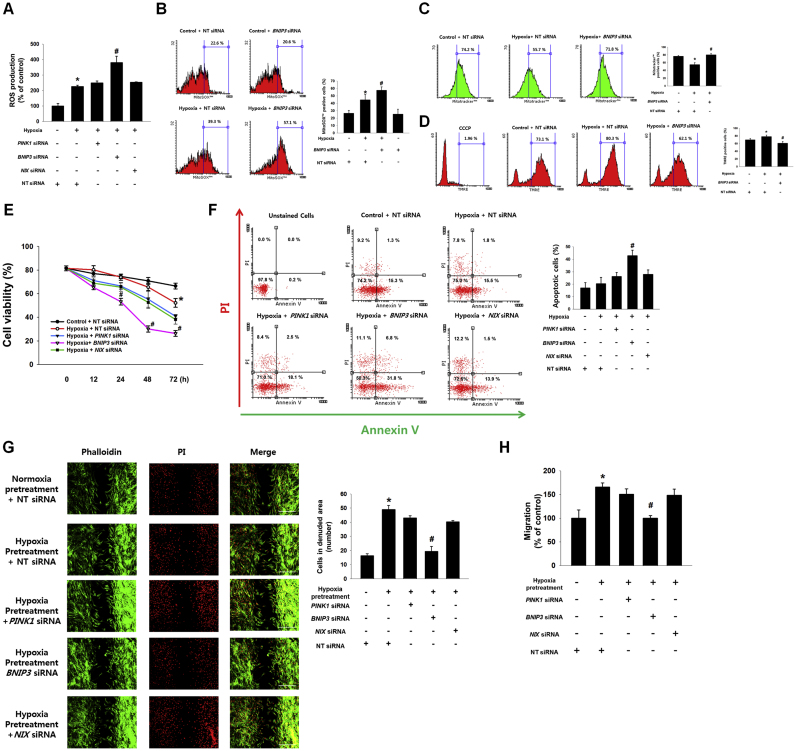
**Role of BNIP3 in the regulation of ROS accumulation and functions in UCB-hMSCs under hypoxia.** (A) *PINK1*, *BNIP3*, *NIX* or NT siRNAs were transfected to UCB-hMSCs prior to hypoxia treatment for 48 h. Intra-cellular ROS level was measured by CM-H_2_DCFDA staining. *n* = *6.* (B-D) *BNIP3* and NT siRNAs were transfected to UCB-hMSCs prior to hypoxia treatment for 48 h MitoSOX™ (B) or Mitotracker™ (C) or TMRE-positive cells (D) which were analyzed by flowcytometer. *n* = *4*. Cells were pretreated 50 μM of CCCP for 2 h (D). (E) UCB-hMSCs transfected with *PINK1*, *BNIP3*, *NIX* or NT siRNAs were exposed to various durations of hypoxia. Cell viability was measured by trypan blue exclusion assay. *n* = *5.* (F) *PINK1*, *BNIP3*, *NIX* or NT siRNAs were transfected to UCB-hMSCs prior to hypoxia treatment for 48 h. Apoptotic cells were detected by annexinV/PI analysis. *n* = *4.* (G) UCB-hMSCs were cultured in ibidi™ insert dish, and siRNAs were transfected to cells prior to hypoxia pretreatment for 24 h. Migrated cells under normoxia for 24 h were visualized by immunostaining with phalloidin and PI. *n* = *4* (magnification, ×100). All scale bars, 200 µm. (H) UCB-hMSC migration was quantified by using luminometer with Oris™ migration assay. *n* = *8.* Quantitative data are presented as a mean ± S.E.M. All confocal images are representative. *p < 0.05 versus control or normoxia pretreatment, *#p* < 0.05 versus hypoxia incubation or hypoxia pretreatment.

Furthermore, we investigated the role of mitophagy regulators induced by hypoxia in ROS production and functions determining therapeutic efficiency of UCB-hMSC transplantation, such as apoptosis, and migration. As shown in Fig. 2A, the intracellular ROS levels in hypoxia-treated UCB-hMSCs, measured by using a general oxidative stress indicator, CM-H_2_DCFDA, increased to 225.5% of the control level, and that of *BNIP3* siRNA-transfected UCB-hMSCs under hypoxia increased further to 380.5% of the control level. Moreover, we performed flow cytometry analysis with MitoSOX™, a mitochondrial ROS-sensitive dye, to determine the role of BNIP3 in mitochondrial ROS production. The number of MitoSOX™-positive cells in *BNIP3* siRNA-transfected UCB-hMSCs under hypoxia was higher than that in hypoxia-pretreated UCB-hMSCs (Fig. 2B). We also assessed the effect of mitophagy regulated by BNIP3 under hypoxia on the uptake of the mitochondrial membrane potential-sensitive fluorescent dye, TMRE. The flow cytometry results with Mitotracker™ and TMRE showed that the decrease in Mitotracker™-positive cells and the increase in TMRE-positive cells by hypoxia were reversed by silencing of BNIP3 expression (Fig. 2C and D). A chemical inhibitor of oxidative phosphorylation, carbonyl cyanide m-chlorophenyl hydrazine (CCCP) was used as a negative control for mitochondrial membrane potential collapse. In addition, we investigated the effect of mitophagy regulators induced by hypoxia on apoptosis and migration in UCB-hMSCs. The cell viability results, measured by trypan blue exclusion assay, showed the viability of UCB-hMSCs at 72 h of hypoxia was lower than that of UCB-hMSCs at 72 h of normoxia, and the viabilities of *BNIP3* siRNA-transfected UCB-hMSCs at 48 and 72 h of hypoxia were significantly lower than those of UCB-hMSCs at 48 and 72 h of hypoxia (Fig. 2E). And, we performed annexinV and PIdouble staining flow cytometry analysis with 48 h of normoxia or hypoxia-treated UCB-hMSCs. Our results showed the number of annexinV-positive apoptotic cells of BNIP3-silenced UCB-hMSCs under hypoxia to be higher than that of UCB-hMSCs under hypoxia (Fig. 2F). To determine the effect of mitophagy regulators induced by hypoxia on UCB-hMSC migration regulated by hypoxia pretreatment, we performed ibidi**™** insert dish and Oris™ migration assays. As shown in Fig. 2G and H, hypoxia pretreatment increased UCB-hMSC migration that had been abolished by *BNIP3* siRNA transfection. These findings suggest that mitophagy induced by BNIP3 is critical for reducing the mitochondrial ROS (mtROS) production, maintaining the mitochondrial membrane potential, and enhancing anti-apoptosis and migration in UCB-hMSC under hypoxia.

### 3.3 Involvement of HIF-1α, FOXO3, and CBP in BNIP3 expression

**Fig. 3:**
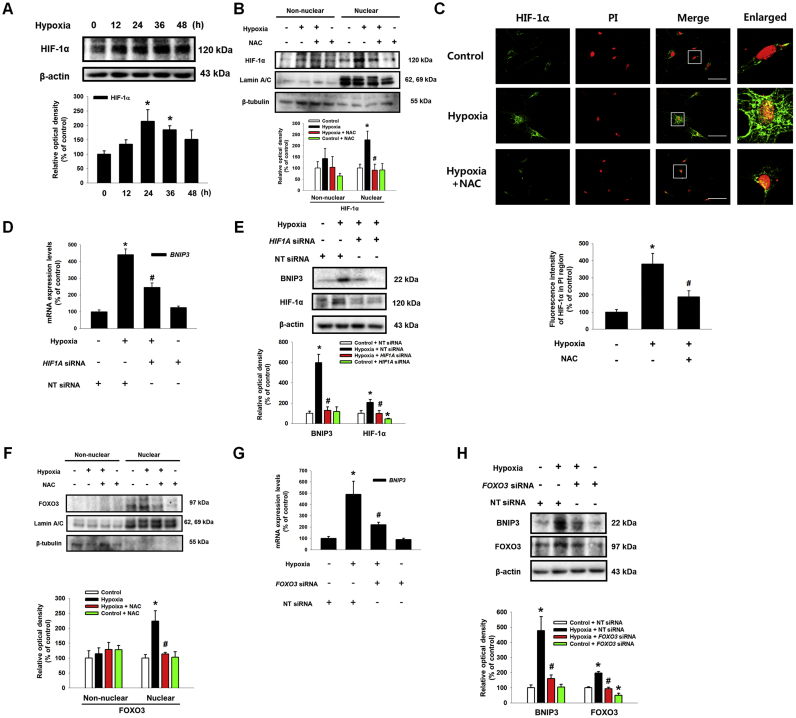
**Role of HIF-1α, FOXO3 in hypoxia-induced BNIP3 expression.** (A) UCB-hMSCs were incubated with various durations of hypoxia (0–48 h). The protein expressions of HIF-1α and β-actin were detected by western blot. *n* = *4.* (B) UCB-hMSCs were pretreated with NAC (5 mM) for 30 min prior to hypoxia incubation for 24 h. The protein expressions of HIF-1α, lamin A/C and β-tubulin in non-nuclear and nuclear fractionized cell samples were assessed by using western blot. *n* = *3.* (C) UCB-hMSCs were immuno-stained with HIF-1α and PI (magnification ×600). Scale bars, 37.5 µm. (D) *HIF1A* siRNA or NT siRNA was transfected to cells prior to hypoxia treatment for 24 h. The mRNA expression of *BNIP3* was analyzed by qPCR. *n* = *6.* (E) The protein expressions of BNIP3 and HIF-1α were detected by western blot. *n* = *4.* (F) NAC (5 mM) was pretreated to UCB-hMSCs prior to hypoxia treatment for 24 h. FOXO3, lamin A/C and β-tubulin proteins expressions were assessed by western blot. *n* = *3.* (G) *FOXO3* siRNA transfected to UCB-hMSCs prior to hypoxia treatment for 24 h. The *FOXO3* mRNA expression was measured by qPCR. *n* = *6.* (H) BNIP3, FOXO3 and β-actin expressions were detected by western blot. *n* = 3. Western blot data were normalized by β-actin, and qPCR data were normalized by *ACTB* mRNA expression level. Lamin A/C and β-tubulin were used as nuclear and non-nuclear protein controls, respectively. Quantitative data are presented as a mean ± S.E.M. All blots and confocal images are representative. *p < 0.05 versus control, *#p* < 0.05 versus hypoxia.

**Fig. 4:**
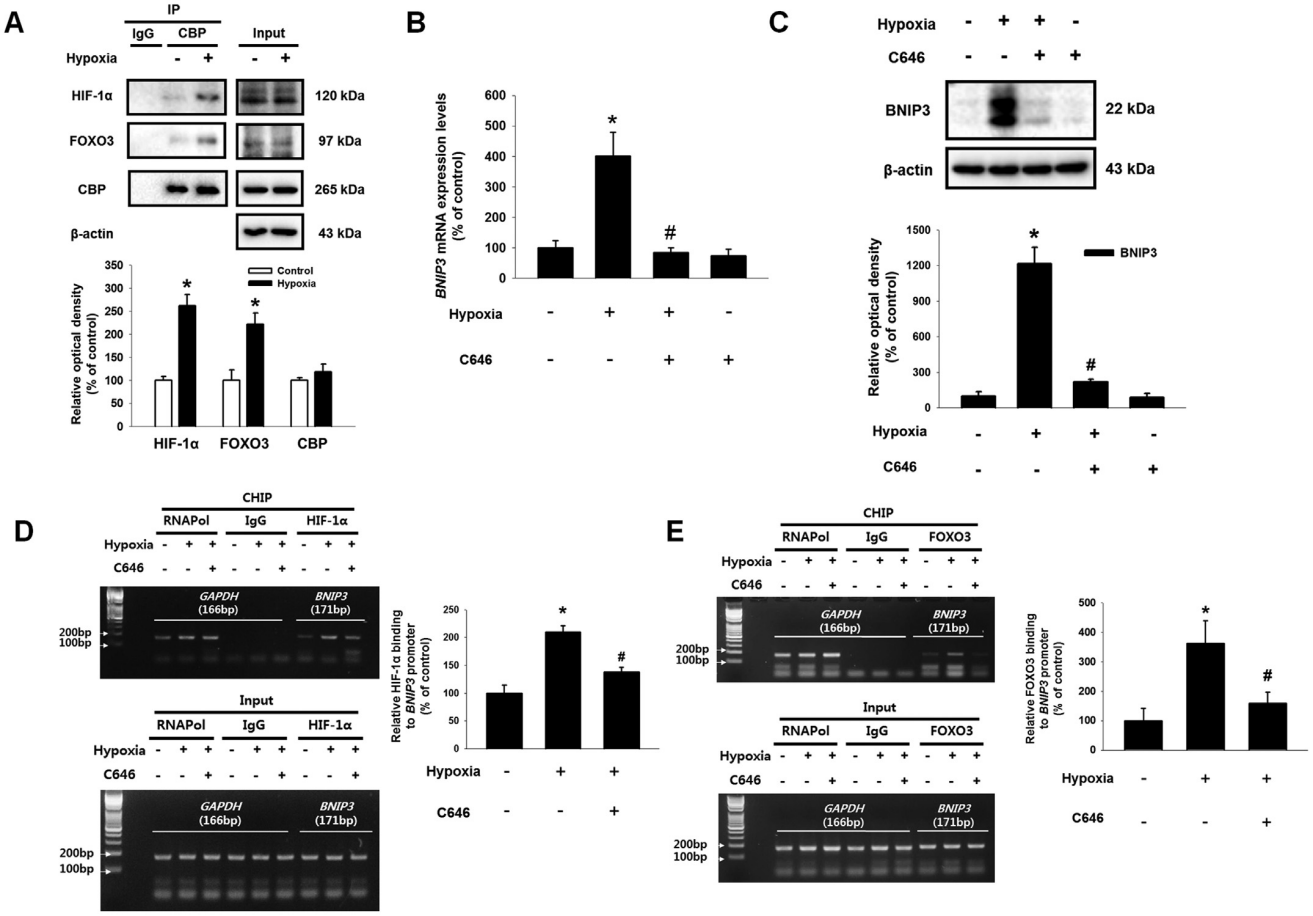
**Involvement of CBP in BNIP3 expression regulated by HIF-1α and FOXO3 under hypoxia. (**A) UCB-hMSCs were incubated with hypoxia condition for 24 h. Co-immunoprecipitation of HIF-1α and FOXO3 with IgG and CBP were shown in left panel. IgG was used as a negative control. The total protein expressions of HIF-1α, FOXO3, CBP and β-actin in lysate were shown in right panel. *n* = *3*. (B) CBP (20 μM) was pretreated to UCB-hMSCs, and cells were incubated with hypoxia for 24 h. The *BNIP3* mRNA expression level was analyzed by qPCR. *n* = *6.* (C) BNIP3 and β-actin protein expressions were analyzed by western blot. Data represent mean ± S.E. *n* = *4.* (D, E) Sample DNA was immuno-precipitated with RNA polymerase, IgG, HIF-1α and FOXO3 specific antibodies. CHIP (top panel) and lysate (bottom panel) samples were amplified with the primers of *GAPDH* and *BNIP3* promoters. Quantitative CHIP data was analyzed by qPCR, and shown in the right panel. *n* = *4.* Western blot data were normalized by β-actin, and qPCR data were normalized by *ACTB* mRNA expression level. Quantitative data are presented as a mean ± S.E.M. All blot images are representative. *p < 0.05 versus control, *#p* < 0.05 versus hypoxia.

HIF-1α and FOXO3 have been well recognized as major transcription factors controlling UCB-hMSC physiology under hypoxia [31], [32]. Although previous investigations have reported that HIF-1α or FOXO3 are involved in BNIP3 expression under hypoxia [33], [34], their roles in BNIP3 expression seem to vary with cell type [35], [36], [37]. To demonstrate the detailed mechanism regulating BNIP3 expression in UCB-hMSCs under hypoxia, we investigated the roles of HIF-1α and FOXO3 in BNIP3 regulation. Then, we confirmed that hypoxia stimulated HIF-1α expression in a time-dependent manner (Fig. 3A). To investigate the role of hypoxia-induced ROS in expressions and activations of HIF-1α and FOXO3, we pretreated a general ROS scavenger, N-acetyl cysteine (NAC) to block the ROS production in UCB-hMSCs under hypoxia. Western blot with nuclear fraction and immunofluorescence results showed ROS produced by hypoxia increased HIF-1α localization in the nucleus (Fig. 3B and C). As shown in Fig. 3D and E, mRNA and protein expressions of BNIP3 were increased by hypoxia but abolished by *HIF1A* siRNA transfection. In addition, we further determined the role of FOXO3 in BNIP3 expression in UCB-hMSCs under hypoxia. Western blot with nuclear fraction results showed ROS production under hypoxia stimulated FOXO3 localization in the nucleus (Fig. 3F). In addition, enhanced BNIP3 expression by hypoxia was partially abolished by silencing of FOXO3 expression (Fig. 3G and H). These results suggest that HIF-1α and FOXO3 induced by ROS production are major factors regulating BNIP3 expression under hypoxia. Subsequently, we observed that hypoxia stimulates the interaction of CBP with HIF-1α and FOXO3 (Fig. 4A). To determine the role of CBP in BNIP3 expression under hypoxia, we pretreated C646, a CBP/p300 inhibitor, to UCB-hMSCs prior to hypoxia treatment. As shown in Fig. 4B and C, mRNA and protein expressions of BNIP3 increased by hypoxia were reversed by C646 pretreatment. Moreover, we investigated the role of CBP in the interaction of HIF-1α and FOXO3 with the *BNIP3* promoter. CHIP assay results showed CBP inactivation by C646 pretreatment interrupted the binding of HIF-1α and FOXO3 to their consensus sequences in the *BNIP3* promoter (Fig. 4D and E). Overall, our results indicate that the interaction of CBP with HIF-1α and FOXO3 stimulated by hypoxia has a critical role in the binding of HIF-1α and FOXO3 to the *BNIP3* gene promoter, leading to transcriptional regulation of BNIP3 expression in UCB-hMSCs.

### 3.4 Role of BNIP3 in FASN-dependent FFA production under hypoxia

**Fig. 5:**
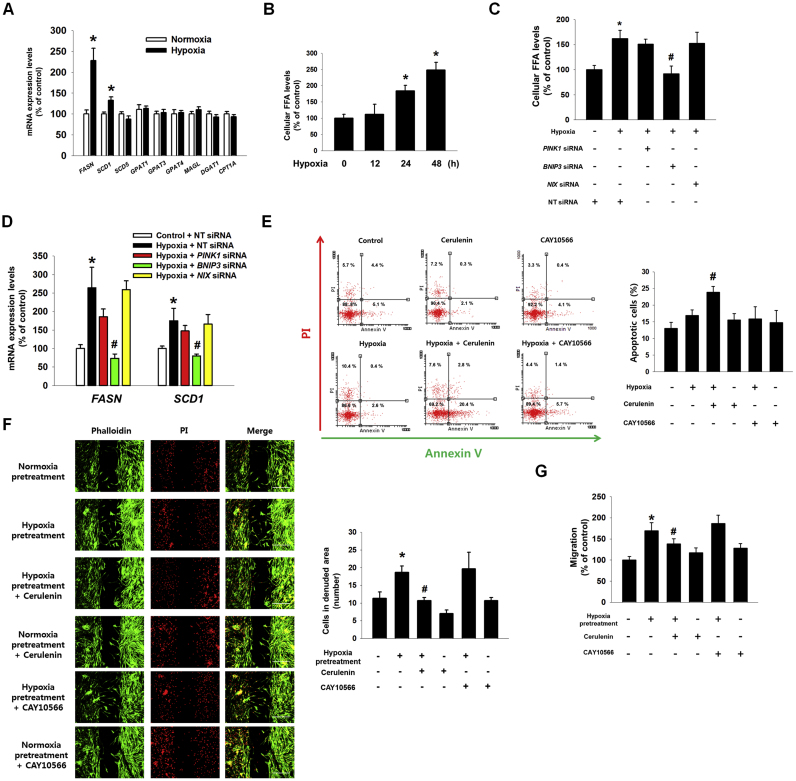
**Regulatory role of hypoxia-induced FASN in UCB-hMSC migration and survival. (**A) UCB-hMSCs were incubated with hypoxia for 24 h. The mRNA expressions of *FASN*, *SCD1*, *SCD5*, *GPAT1*, *GPAT3*, *GPAT4*, *MAGL*, *DGAT1 and CPT1A* were analyzed by qPCR. *n* = *5.* (B) UCB-hMSCs were treated with various durations of hypoxia (0–48 h). Cellular FFA level was measured with FFA detection kit. *n* = *6.* (C) Cells were transfected with *PINK1*, *BNIP3*, *NIX* or NT siRNAs, treated with hypoxia for 24 h. Cellular FFA level was shown. *n* = *6*. (D) The *FASN* and *SCD1* mRNAs expressions level were assessed by qPCR. *n* = *6.* (E) Cells were pretreated with cerulenin (10 μM) or CAY10566 (100 μM) for 30 min prior to hypoxia for 48 h. Apoptotic cells were detected by annexinV/PI analysis. *n* = *4.* (F) Cells were plated in ibidi™ insert dish, and cerulenin (10 μM) or CAY10566 (100 μM) were pretreated to cells for 30 min prior to hypoxia pretreatment for 24 h. Migrated cells under normoxia for 24 h were visualized by immunostaining with phalloidin and PI. *n* = *4* (magnification, ×100). Scale bars, 200 µm. (G) Cell migration was measured by Oris™ migration assay. *n* = *8.* The mRNA expression level was normalized with *ACTB* mRNA expression level. The quantitative data are presented as a mean ± S.E.M. All confocal images are representative. *p < 0.05 versus control or normoxia pretreatment, *#p* < 0.05 versus hypoxia incubation or hypoxia pretreatment.

To confirm the role of BNIP3 expression induced by hypoxia in lipid metabolism, we examined mRNA expressions of lipid metabolic enzymes including *FASN*, stearoyl-CoA desaturase 1 (*SCD1*), *SCD5*, glycerol-3-phsphate acyltransferase 1 (*GPAT1*), *GPAT3*, *GPAT4*, monoacylglycerol lipase (*MAGL*), diglyceride acyltransferase 1 (*DGAT1*), and carnitine palmitoyltransferase 1A (*CPT1A*) in UCB-hMSCs under hypoxia. As shown in Fig. 5A, we observed an increase in FFA-producing enzymes including *FASN* and *SCD1*. Particularly, mRNA expression of *FASN* in UCB-hMSCs under hypoxia increased to 225.3% of the control level. We further confirmed that hypoxia stimulated production of cellular FFA in UCB-hMSCs after 24 h and 48 h (Fig. 5B). In addition, up-regulated cellular FFA production and mRNA expressions of *FASN* and *SCD1* by hypoxia were reversed by silencing BNIP3 expression (Fig. 5C and D). Furthermore, we determined the role of FASN and SCD1 regulated by BNIP3 in apoptosis and migration of UCB-hMSCs under hypoxia. AnnexinV and PI double staining flow cytometry results showed that FASN inhibition by cerulenin (a FASN inhibitor) but not by CAY10566 (a SCD1 inhibitor) increased apoptosis of UCB-hMSC exposed to hypoxia (Fig. 5E). There was no significant difference between groups treated with normoxia or hypoxia. The results of ibidi™ insert dish and Oris™ migration assays showed that hypoxia pretreatment stimulated UCB-hMSC migration, abolished by pretreatment of cerulenin, but not by CAY10566 (Fig. 5F and G). These results suggest that FASN-dependent FFA production is important for the regulation of apoptosis and migration in UCB-hMSCs under hypoxia.

**Fig. 6:**
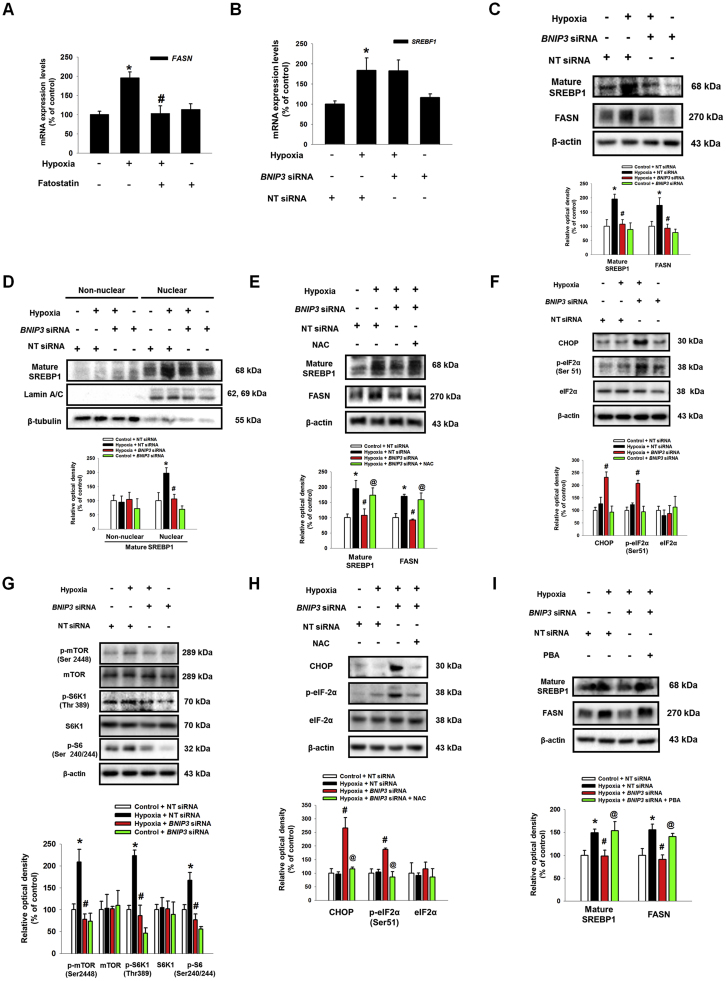
**The regulatory role of ER stress induction by BNIP3 knock down in SREBP1 and FASN expressions. (**A) UCB-hMSCs were pretreated with fatostatin (10 μM) for 30 min prior to hypoxia treatment for 24 h. The mRNA expression of *FASN* was analyzed by qPCR. *n* = *6*. (B) UCB-hMSCs were transfected with *BNIP3* or NT siRNAs, incubated with hypoxia for 24 h. The mRNA expression of *SREBF1* was measured by qPCR. *n* = *6.* (C) The protein expressions of mature SREBP1, FASN, BNIP3 and β-actin were detected by western blot. *n* = *4.* (D) Cells were transfected with NT or *BNIP3* siRNA for 24 h prior to hypoxia incubation for 48 h. Mature SREBP1, lamin A/C and β-tubulin expressions in non-nuclear and nuclear fractionized samples were detected by western blot. *n = 3.* (E) *BNIP3* or NT siRNAs-transfected UCB-hMSCs were pretreated with NAC (500 μM) prior to hypoxia treatment for 48 h. Mature SREBP1, FASN, BNIP3, and β-actin were detected by western blot. *n* = *3.* (F, G) UCB-hMSCs were transfected with *BNIP3* or NT siRNAs, incubated with hypoxia for 48 h. CHOP, p-eIF2α (Ser51), eIF2α, p-mTOR (Ser2448), mTOR, p-S6K1 (Thr389), S6K1, p-S6 (Ser240/244) and β-actin expressions were shown. *n* = *4.* (H) *BNIP3* or *NT* siRNAs-transfected UCB-hMSCs were pretreated with NAC (500 μM) for 1 h prior to hypoxia treatment for 48 h. The expressions of CHOP, p-eIF2α (Ser51), eIF2α and β-actin were detected by western blot. *n* = *4.* (I) Cells were transfected with *BNIP3* or NT siRNAs, pretreated with PBA (100 μM) for 1 h prior to hypoxia treatment for 48 h. Mature SREBP1, FASN and β-actin proteins expressions were shown. Western blot data were normalized by β-actin, and qPCR data were normalized by *ACTB* mRNA expression level. Lamin A/C and β-tubulin were used as nuclear and non-nuclear protein controls, respectively. The quantitative data are presented as a mean ± S.E.M. All blot images are representative. *p < 0.05 versus control, *#p* < 0.05 versus hypoxia, *@p* < 0.05 versus *BNIP3* siRNA-transfected UCB-hMSCs with hypoxia.

A SREBP1 has been reported as a major transcription factor regulating FASN expression in hypoxia [38]. To confirm the role of SREBP1 in hypoxia-induced FASN, we pretreated fatostatin, a SREBP1 inhibitor. As shown in the Fig. 6A, up-regulation of *FASN* mRNA expression by hypoxia was abolished by pretreatment of fatostatin. Next, we checked the effect of BNIP3 silencing on expression of mature SREBP1 (68 kDa) in UCB-hMSCs under hypoxia to identify the mechanism involved in how BNIP3 induced by hypoxia regulates FASN expression. Interestingly, our data showed that mRNA expression of *SREBF1* increased by hypoxia was not affected by *BNIP3* siRNA transfection (Fig. 6B). However, the protein expressions of mature SREBP1 and FASN were inhibited by *BNIP3* siRNA transfection (Fig. 6C). In addition, silencing of BNIP3 expression also inhibited translocation of mature SREBP1 into the nucleus (Fig. 6D). There were no significant differences in HIF-1α expressions between groups treated with hypoxia or *BNIP3* siRNA (Sup. Fig. S3). These results suggest BNIP3 silencing-regulated SREBP1 expression is transcription independent. We hypothesized excessive ROS production by BNIP3 silencing may be a regulator which leads to suppression of SREBP1/FASN pathway. To determine the effect of excessive ROS production by BNIP silencing on endoplasmic reticulum (ER) stress, such as eIF-2α and CHOP, expressions of mature SREBP1 and FASN, therefore, we pretreated low dose of NAC to BNIP3-silenced UCB-hMSCs prior to hypoxia treatment; the results confirmed that excessive ROS production induced by *BNIP3* siRNA transfection was decreased by low dose NAC pretreatment (Sup. Fig. S4). As shown in the Fig. 6E, down-regulated protein expressions of mature SREBP1 and FASN were recovered by NAC pretreatment. In addition, BNIP3 silencing further increased CHOP expression and eIF-2α phosphorylation, and decreased mTOR, S6K1, and S6 phosphorylations in UCB-hMSC under hypoxia (Fig. 6F and G). Low dose NAC pretreatment abolished augmentation of CHOP expression and eIF2α phosphorylation by BNIP3 silencing (Fig. 6H). Next, we pretreated a ER stress inhibitor PBA to *BNIP3* siRNA-transfected UCB-hMSCs under hypoxia to confirm the effect of ER stress augmentation on mature SREBP1 and FASN expression. And, we observed that suppressed expressions of mature SREBP1 and FASN were recovered by PBA pretreatment (Fig. 6I). Taken together, our results suggest that the reduction of ROS by BNIP3 in hypoxia induces SREBP1 maturation and FASN expression through the suppression of ER stress and activation of mTOR/S6K1/S6 pathway.

### 3.5 Effect of BNIP3 on therapeutic potential of UCB-MSCs under hypoxia

**Fig. 7:**
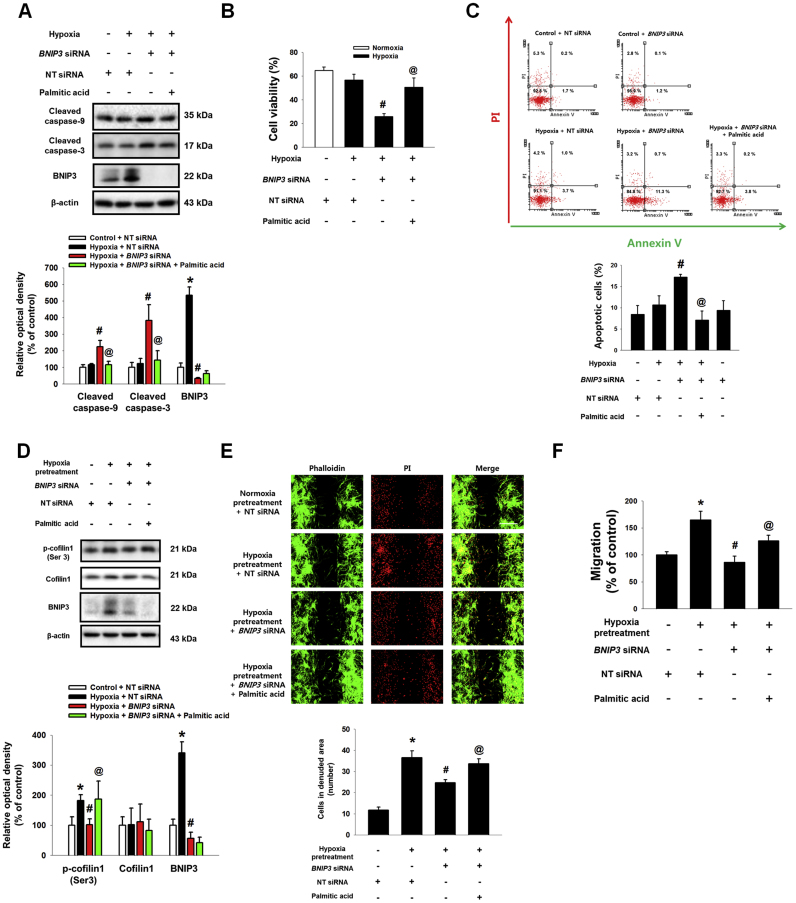
**The protective effect of PA in BNIP3-silenced UCB-hMSC migration and survival. (**A) The PA (20 μM) pretreated to UCB-hMSC transfected with *BNIP3* or NT siRNAs, and then cells were incubated with hypoxia for 48 h. The expressions of cleaved caspase-9, cleaved caspase-3, BNIP3 and β-actin were analyzed by western blot. *n* = *4.* (B) Cell viability was measured by trypan blue exclusion assay. *n* = *6.* (C) The percentage of apoptotic cells were assessed by AnnexinV/PI staining. *n* = *4.* (D) Cells transfected with *BNIP3* or NT siRNAs were pretreated with PA (20 μM) prior to hypoxia pretreatment for 24 h. And, then cells were incubated with normoxia for 24 h. The expressions of p-cofilin1 (Ser3), cofilin1, BNIP3 and β-actin were detected by western blot. *n* = *4.* (E) Migrated UCB-hMSCs were visualized by immunostaining with phalloidin and PI. *n* = *4* (magnification, ×100). Scale bars, 200 µm. (F) Cell migration was assessed by using luminometer with Oris™ migration assay. *n* = *8.* Western blot data were normalized by β-actin. The quantitative data are presented as a mean ± S.E.M. All blot images and confocal images are representative. *p < 0.05 versus control or normoxia pretreatment, *#p* < 0.05 versus hypoxia or hypoxia pretreatment, *@p* < 0.05 versus *BNIP3* siRNA-transfected UCB-hMSC with hypoxia or hypoxia pretreatment.

Our previous study reported that PA produced by FASN is a major FFA regulating hypoxia-induced UCB-hMSC functions [6]. Therefore, we investigated the effect of PA, a FASN major lipid metabolite, on apoptosis and migration to confirm the role of BNIP3-induced FASN in UCB-hMSC function under hypoxia. As shown in Fig. 7A, protein expressions of cleaved caspase-3 and cleaved caspase-9 in UCB-hMSCs under hypoxia were increased by *BNIP3* siRNA transfection but decreased by PA pretreatment. Trypan blue exclusion cell viability assay and annexinV/PI double staining flow cytometry results showed that the decrease in cell viability and increase in the number of apoptotic cells by BNIP3-silenced UCB-hMSCs under hypoxia were reversed by PA pretreatment (Fig. 7B and C). Moreover, we found that BNIP3 silencing did not increase the number of apoptotic of UCB-hMSCs under normoxia (Fig. 7C). Next, we also investigated the effect of BNIP3 silencing and PA on F-actin polymerization regulatory protein. As shown in Fig. 7D, phosphorylation of cofilin-1 at Ser3 residue was up-regulated by hypoxia pretreatment, but abolished by *BNIP3* siRNA transfection. PA pretreatment recovered the inhibitory effects of BNIP3 silencing in terms of cofilin-1 phosphorylation in UCB-hMSCs (Fig. 7D). Consistent with those results, the ibidi™ insert dish and Oris™ migration assay results showed that down-regulated migration of BNIP3-silenced UCB-hMSCs by hypoxia pretreatment was partially recovered by PA pretreatment (Fig. 7E and F). Our results indicate that exogenous PA rescues reduction of survival and migration by BNIP3 silencing in UCB-hMSCs under hypoxia.

**Fig. 8:**
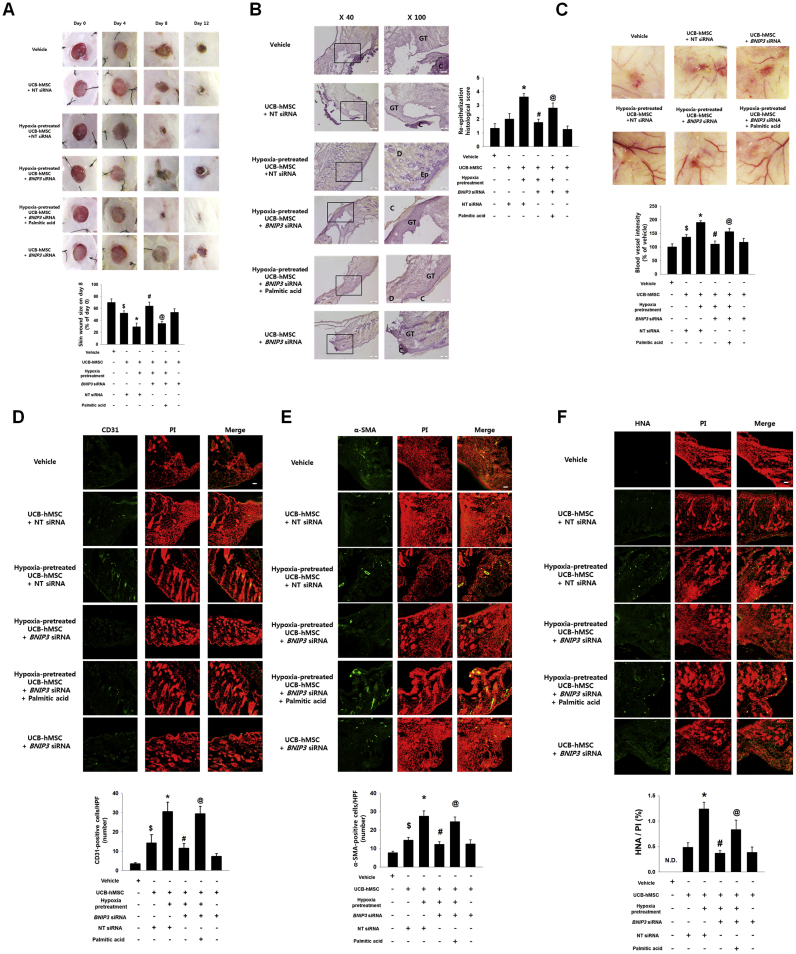
**The role of PA in BNIP3 silenced UCB-hMSC survival in the mouse skin wound healing model.** (A) Mouse skin wound surgery with UCB-hMSC transplantation was performed as described in Section 2. Representative gross wound images were acquired at post injection days 0, 4, 8, 12. Skin wound sizes at day 8 were compared with wound size at day 0. *n* = *5.* (B) Tissue slide samples were stained with hematoxylin and eosin. Low and high magnified histological gross images are shown in the left and right panels, respectively. Scale bars, 260 µm (magnification, ×40) and 100 µm (magnification, ×100). *n* = *5.* (C) Representative images of blood vessels in skin wounds on day 12 (top panel). Vessel density was analyzed by using ImageJ program (bottom panel). *n* = *5.* (D-F) Histological tissue samples were immuno-stained with CD31, α-SMA, and HNA-specific antibodies and PI for counterstaining. α-SMA and HNA-positive cells were visualized by confocal microscopy. The number of CD31 and α-SMA-positive cells in high power field (HPF), and the percentage of HNA-positive cells in total cells were analyzed by using Metamorph software. Scale bars, 100 µm (magnification, ×100). *n* = *5.* Data are presented as a mean ± S.E.M. *$p* < 0.05 versus vehicle group, *p < 0.05 versus UCB-hMSC group given NT siRNA, *#p* < 0.05 versus UCB-hMSC group given NT siRNA with hypoxia pretreatment, *@p* < 0.05 versus UCB-hMSC group given *BNIP3* siRNA with hypoxia pretreatment.

Furthermore, we confirmed the effect of BNIP3 expression regulated by hypoxia pretreatment and PA on mouse skin wound healing with UCB-hMSC transplantation. 8 days after skin wound surgery, the wound area of mice was reduced to a significantly great extent in UCB-hMSCs with hypoxia. In addition, the wound area of mice given *BNIP3* siRNA-silenced UCB-hMSCs with hypoxia pretreatment was larger than that of mice given UCB-hMSCs with hypoxia or *BNIP3* siRNA-transfected UCB-hMSCs with pretreatments of hypoxia and PA (Fig. 8A). Meanwhile, our results showed that there was no statistical significance of the difference between wound area of mice given UCB-hMSCs and that of mice given *BNIP3* siRNA-transfected UCB-hMSCs. A histological evaluation at 12 days after skin wound surgery showed the wound bed to be completely covered by transplantations of UCB-hMSCs with hypoxia pretreatment and of *BNIP3* siRNA-transfected UCB-hMSCs with pretreatments of hypoxia and PA (Fig. 8B). In addition, the transplantation of UCB-hMSCs with hypoxia pretreatment showed re-epithelization as well as wound closure at the wound site. The re-epithelization histological scoring was quantified according to the criteria provided in Supplementary Table S1. The re-epithelization histological scoring results showed that the re-epithelization score of transplantation of hypoxia-pretreated UCB-hMSCs is the highest among all experimental groups. Re-epithelization score of the mice given *BNIP3* siRNA-transfected UCB-hMSCs with hypoxia and PA was higher than that of mice given *BNIP3* siRNA-transfected UCB-hMSCs with hypoxia pretreatment. There was no statistical significance between groups treated with vehicle or UCB-hMSCs alone. We further investigated the effect of BNIP3 regulation by pretreatment of either hypoxia or PA on angiogenesis capacity and survival of transplanted UCB-hMSCs. Our results showed that transplantation of UCB-hMSCs with hypoxia pretreatment increased blood vessel formation and the amount of pan-endothelial marker CD31, myofibroblast marker α-SMA-positive and HNA-positive cells at the wound site compared to transplantation of UCB-hMSCs alone. In addition, angiogenesis capacity and survival rate of *BNIP3* siRNA-transfected UCB-hMSCs with hypoxia pretreatment was lower than those of UCB-hMSCs with hypoxia pretreatment and *BNIP3* siRNA-transfected UCB-hMSCs with hypoxia and PA (Fig. 8C–F). Overall, these findings indicate that decreased wound healing capacity of hypoxia-pretreated UCB-hMSCs by BNIP3 silencing is recovered by PA pretreatment.

## 4 Discussion

The present study highlights the mechanism controlling mitophagy in hypoxia and the relevance of mitophagy in the regulation of lipid metabolism and therapeutic functions, such as apoptosis, migration, and wound repair of UCB-hMSCs under hypoxia. Although PINK1 and NIX were increased by hypoxia, our results suggest that BNIP3 is a major mitophagy regulator stimulated by hypoxia in UCB-hMSCs, and it has a critical role in regulation of UCB-hMSC functions. There are several previous reports showing mitophagy induced by hypoxia via PINK1, NIX, BNIP3, and FUNDC1, but which factor is important for hypoxia-induced mitophagy appeared to be cell type-specific [39], [40]. Interestingly, our results showed hypoxia suppressed FUNDC1 expression. Although several investigators reported that FUNDC1 is associated with hypoxia-induced mitophagy, the effect of hypoxia on FUNDC1 expression is controversial, and the exact mechanism by which hypoxia regulates FUNDC1 expression has not been fully described [11], [39], [41]. BNIP3 has been known as a non-selective mitophagy regulator removing healthy and unhealthy mitochondria mediated by interaction of the LC3 interacting region motif with LC3 [42]. Consistent with previous results, our current results showed an increase in co-localization of COX4- and BNIP3-positive regions with LC3B. We also presented results showing that BNIP3 controls mitochondrial quality through the regulation of mtROS and mitochondrial membrane potential in hypoxia. It has been reported that mitophagy induced by BNIP3 and NIX removes damaged mitochondria and protects against ROS accumulation [43], [44]. In addition, dysregulation of the redox system and ROS accumulation directly link to stem cell apoptosis [1]. The role of BNIP3 in cell death is debated, although it is reported that BNIP3 is a BH-3 only protein like other pro-apoptotic BCL-2 family members. Many researchers have reported that BNIP3 contributes to hypoxia-induced cell death through various mechanisms [45], [46]. But, there is another report showing a protective effect of BNIP3 induction [47]. Consistent with those findings, our results showed silencing of BNIP3 induced UCB-hMSC apoptosis. Therefore, further investigations using various types of stem cells are required to obtain more complete elucidation of the role of BNIP3 in stem cell functions under hypoxia.

Present study focused on identifying the molecular mechanism involved in how HIF-1α and FOXO3 contribute to BNIP3 transcription in UCB-hMSCs under hypoxia. Our results showed both HIF-1α and FOXO3 expressions up-regulated by hypoxia contribute to BNIP3 transcription. Notably, BNIP3 expression induced by hypoxia was mostly suppressed by *HIF1A* siRNA transfection, but also partially suppressed by *FOXO3* siRNA transfection. These results suggest the possibility of HIF-1α acting as an upstream regulator of FOXO3 in terms of BNIP3 regulation under hypoxia. Previous studies investigating the mechanism of FOXO3 regulation by hypoxia demonstrated that HIF-1α induced by oxidative stress stimulates FOXO3 expression [32], [37]. In addition, it has been reported that cooperation of HIF-1α with FOXO3 is required for high-level transcription of *BNIP3* mRNA by hypoxia [48]. We hypothesized that there may be a molecule that mediates their cooperative action on BNIP3 expression. In addition, our results demonstrated that CBP acts as a transcriptional co-activator interacting with HIF-1α and FOXO3 in the nucleus, and is closely associated with the binding of HIF-1α and FOXO3 to the *BNIP3* gene promoter region indicating an epigenetic action of CBP for *BNIP3* mRNA transcription. CBP/p300 has recruitment sites for physical interaction with various transcription factors including HIF-1α and FOXOs [49], [50]. Acetylation of histone by CBP/p300 neutralizes the positive charge of lysine residue, leading to an increase in the DNA accessibility of transcription factors [51], [52]. Moreover, CBP/p300 directly increases HIF-1α stability and FOXO3 activity through acetylation [53], [54], [55]. Taken together, we propose that CBP induces transcriptional synergism between HIF-1α and FOXO3; an effect that is required for BNIP3 expression under hypoxia.

Our present study highlights a critical role for the BNIP3 as a lipid metabolism regulator in UCB-hMSCs under hypoxia. Based on our results, hypoxia-induced BNIP3 regulates mRNA expressions of *FASN* and *SCD1*, which are involved in de novo synthesis of saturated and unsaturated long-chain FFAs. We used cerulenin and CAY10566 to distinguish the role of FASN and SCD1 regulated by BNIP3 in migration induced by hypoxia pretreatment and survival under hypoxia in UCB-hMSCs. Intriguingly, our results showed that migration and survival of UCB-hMSCs under hypoxia are regulated by FASN, not by SCD1. There have been several previous reports investigating the role of lipogenesis-regulating enzymes in stem cell regulation. For example, FASN-dependent lipogenesis is highly active in adult neural stem and progenitor cells that are associated with adult neurogenesis [56]. FASN inhibition by cerulenin suppressed proliferation and migration as well as stemness marker expression in glioma stem cell [57]. In addition, SCD1 has a tumor suppressive role in survival of leukemia stem cells and eliminates undifferentiated tumorigenic pluripotent stem cells [58], [59]. Taken together, those findings suggest that different stem cell types have different levels of sensitivity to saturated and unsaturated long-chain FFAs. However, further investigation of the role of SCD1 up-regulation by hypoxia in the stem cell is required to provide insight into lipid metabolism of stem cells under hypoxia. Meanwhile, there is a previous study reporting that the loss of BNIP3 increased FASN expression and lipid synthesis in hepatocytes due to ROS accumulation [60], which is inconsistent with our results. The difference between results is believed to be due to cell type and physiological condition differences like degree of ROS accumulation. Indeed, present study suggested both hypoxia and BNIP3 silencing stimulate ROS accumulation, but BNIP3 silencing caused severe ROS accumulation, which may lead to suppression of anti-apoptosis, migration and FFA production.

Our results further indicate that the regulatory role of BNIP3 in ROS accumulation is associated with SREBP1/FASN pathway dependent lipogenesis in UCB-hMSCs under hypoxia. There are several reports showing the mechanism of regulation of SREBP1 expression. Previous studies suggested that SREBP1 expression is regulated by HIF-1α and mTORC1 [61], [62]. In addition, we previously demonstrated that SREBP1 expression is mainly induced by HIF-1α as an up-stream regulator, but not mTOR, in UCB-hMSCs under hypoxic condition [6]. SREBP1 consists of two isoforms of *SREBF1* gene, such as SREBP1a and SREBP1c. Previous reports presented that SREBP1c expression is responsible under hypoxic condition, which is associated with de novo lipogenesis [63], [64]. Although both SREBP1a and SREBP1c are involved in lipid metabolism [65], [66], most of previous studies present a SREBP-1c as a major regulator of FASN expression [67], [68]. Those previous findings indicate that SREBP-1c is a major regulator for FASN-induced lipogenesis in UCB-hMSCs under hypoxia.

Present study showed that the excessive ROS production by BNIP3 silencing decreased SREBP1 and FASN expressions. We pretreated NAC to suppress the ROS production potentiated by BNIP3 silencing. In our results, NAC pretreatment increased SREBP1 and FASN expressions reduced by BNIP3 silencing, and decreased ER stress markers expressions potentiated by BNIP3 silencing. In particular, we observed that BNIP3-silenced UCB-hMSCs under hypoxia still have high levels of HIF-1α protein and *SREBF1* mRNA, although BNIP3 silencing suppressed expressions of mature SREBP1 and FASN as well as FFA production. Furthermore, we observed that augmentation of ER stress by oxidative stress significantly suppressed SREBP1 and FASN expressions. Two mechanisms involved in how hypoxia induces ER stress have been reported; one is driven by downregulation of Ero1 oxidase and the other involves induction of PERK signaling by GSK-3β activated by oxidative stress [69], [70]. Previous reports demonstrated that the chronic and excessive ER stress inactivates mTORC1 signaling, which leads to translational inhibition [71], [72]. Indeed, we confirmed suppression of hypoxia-activated mTORC1 signaling in BNIP3-silenced UCB-hMSCs. Taken together, we present that NAC prevents against the ER stress-induced translational inhibition by BNIP3 silencing, which leads to induction of SREBP1 and FASN expressions.

There is ample evidence showing that enhancement of migration and anti-apoptosis is a potential therapeutic strategy in regenerative medicine using MSCs [73], [74]. Our study results indicate PA, a major product of FASN, is a crucial lipid metabolic factor involved in UCB-hMSC migration and anti-apoptosis. Previous reports showed that PA stimulates migration through plasminogen activator inhibitor-1 expression and F-actin rearrangement mediated by Cdc42 [75], [76]. Our previous and present results demonstrate that enhancement of migratory ability of UCB-hMSCs under hypoxia is involved in F-actin polymerization via cofilin-1 phosphorylation and RhoA activation, not via Rac1 and Cdc42 [6]. Meanwhile, several investigators have reported on the effect of PA on cell survival, and it seems to be dose-dependent [77], [78]. Although our present study showed PA pretreatment recovers UCB-hMSC apoptosis under hypoxia, additional investigation revealing the mechanism involved in PA regulation of apoptosis of UCB-hMSCs under hypoxia is needed. It is clear that activation of GPR40 has key roles in calcium influx, thereby inducing a cAMP level increase, which drives anti-apoptotic effects [79]. Furthermore, we present in vivo evidence that BNIP3-induced UCB-hMSCs transplantation by hypoxia pretreatment or PA accelerates myoblast switch and blood vessel formation, as well as the skin wound healing process. A phenotypic switch of fibroblasts toward myofibroblasts via myofibroblast development has been reported as a representative characteristic of wound repair and maturation [80], [81]. Although transplanted UCB-hMSCs at the wound site are exposed to hypoxia, due to low density of vascular distribution, BNIP3 silencing of UCB-hMSCs with normoxia pretreatment did not significantly affect this process. It is implied that BNIP3 may contribute to the survival of transplanted UCB-hMSCs in the initial wound healing phase. In addition, we observed that PA recovers the reduced regenerative capacity and survival of BNIP3-silenced UCB-hMSCs with hypoxia pretreatment. It has been shown that the tissue regenerative effect associated with transplantation of MSCs is induced by a paracrine effect rather than by cell replacement, and dynamic migration of transplanted cells into the wound site has been correlated with repair process initiation and the immune modulatory process [82], [83]. Consequently, these results indicate that BNIP3 expression and PA production induced by hypoxia have critical roles in the regulation of migration and survival in transplanted UCB-hMSCs, thereby determining the therapeutic efficacy of UCB-hMSC transplantation with hypoxia pretreatment. There are many reports showing the effect of PA in cell functions, but it appears dose-dependent [6], [84], [85]. A previous report showed high concentration of PA (above 100 μM) induced cell death in a dose-dependent manner [86]. Transplanted cells into the healthy or obese or diabetes patients are exposed to high PA (120–220 μM) microenvironment compared to in vitro culture medium which does not contain PA [87], [88]. Based upon those findings, we proposed that high PA microenvironment in obese or diabetic patients may decrease therapeutic efficiency of UCB-hMSCs transplantation. However, our previous report presented that PA pretreatment increased proliferation and migration via activation of mTORC1 signaling in UCB-hMSCs under hypoxia [6]. Therefore, we provide PA as an inducer or a signal molecule for enhancing the therapeutic potential of cell prior to UCB-hMSCs transplantation.

**Fig. 9:**
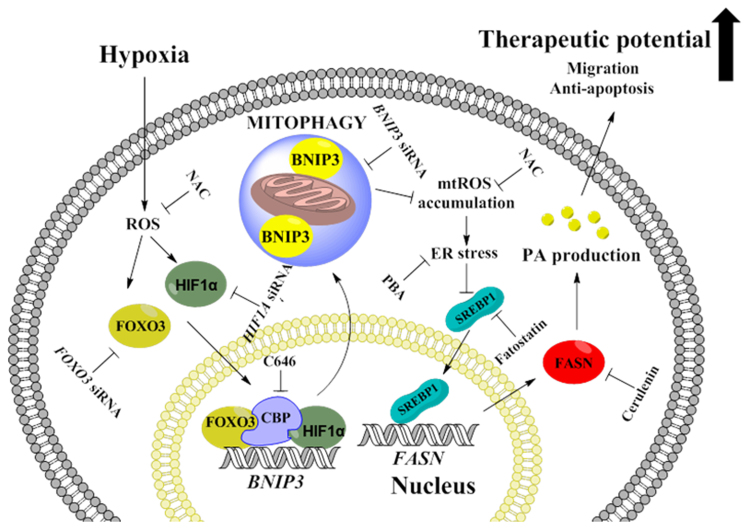
**The schematic model for mechanism involved in the role of BNIP3 induced by hypoxia in UCB-hMSC therapeutic potential.** Hypoxia stimulates BNIP3 expression through the formation of HIF-1α, FOXO3, CBP complex in the nucleus leading to BNIP3-dependent mitophagy. BNIP3 silencing induces accumulation of mitochondrial ROS (mtROS), associated with ER stress augmentation. Augmented ER stress suppresses SREBP1 protein expression and nuclear translocation followed by inhibition of FASN-mediated PA production. PA rescues decreased therapeutic potential including migration and anti-apoptosis by BNIP3 silencing in UCB-hMSCs under hypoxia.

In conclusion, our investigation has demonstrated hypoxia-stimulated BNIP3-mediated mitophagy occurs via ROS-dependent HIF-1α and FOXO3 activation, which are critical for lipogenesis regulated by the SREBP1/FASN pathway and leading to migration, anti-apoptosis, and wound healing in UCB-hMSCs (Fig. 9). To our knowledge, this is the first identification of a major mitophagy regulator involved in lipid metabolism alteration of UCB-hMSCs under hypoxia. Moreover, we suggest a BNIP3 as a novel target for control of lipid metabolism and enhancement of therapeutic potential in UCB-hMSCs.

## Author contribution

Lee.HJ: Conception and design, Collection and/or assembly of data, Data analysis and interpretation, Manuscript writing.

Jung.YH: Data analysis and interpretation, Manuscript writing.

Choi.GE: Collection of data.

Ko.SH: Collection of data.

Lee.S: Data analysis and interpretation.

Lee.SH: Conception and design.

Han.HJ: Conception and design, Data analysis and interpretation, Manuscript writing.

## Conflict of interest

The authors declare no conflict of interest.
