# Supplementary material for: BNIP3 induction by hypoxia stimulates FASN-dependent free fatty acid production enhancing therapeutic potential of umbilical cord blood-derived human mesenchymal stem cells
Source: Redox Biol. 2017 Jul 4;13:426–43. doi: 10.1016/j.redox.2017.07.004 (PMC5508529; doi:10.1016/j.redox.2017.07.004)
Supplement: Supplementary file 1 — Supplementary material [file mmc1.docx]

**Supplementary Figures**


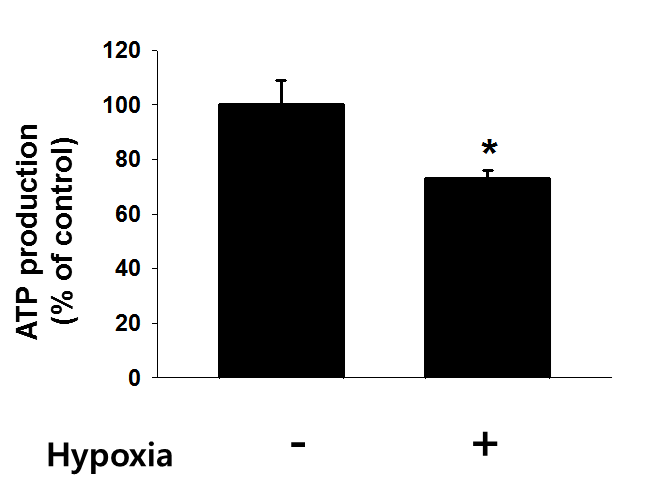


**Supplementary Figure S1. The effect of hypoxia in ATP production.** UCB-hMSCs were treated with hypoxia for 24h. Cellular ATP level is measured by using commercial ATP detection kit described in Materials & Methods section. Data are showed as a mean ± S.E.M. *n=5*.


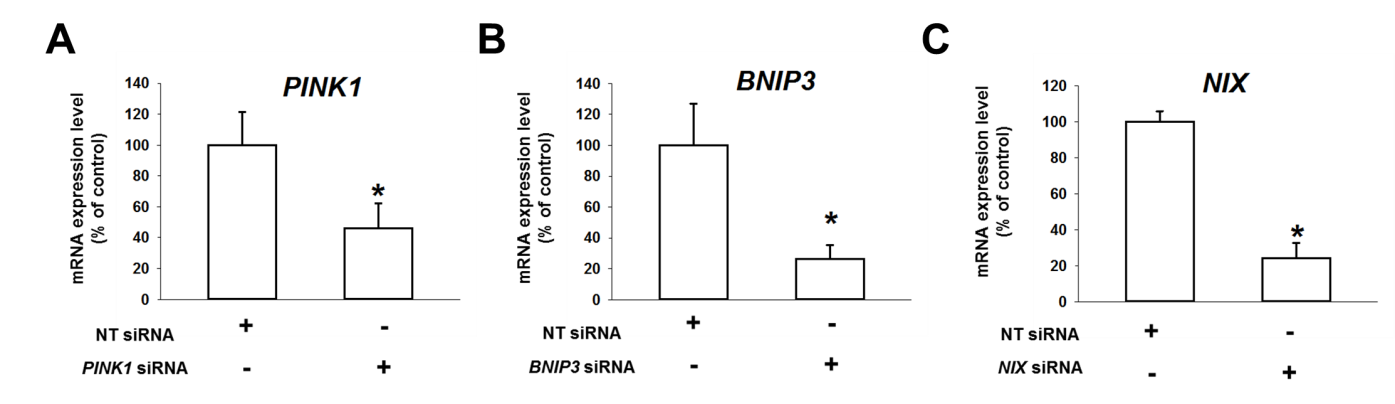


**Supplementary Figure S2. The effect of siRNAs on mRNA expressions of *PINK1*, *BNIP3* and *NIX*.** A-C UCB-hMSCs were transfected with *PINK1*, *BNIP3* and *NIX* siRNAs for 24 h. The mRNA expressions of *PINK1*, *BNIP3* and *NIX* were analyzed by qPCR. The mRNA expression levels were normalized with *ACTB* mRNA expression level. Data are presented as a mean ± S.E.M. *n = 4.*


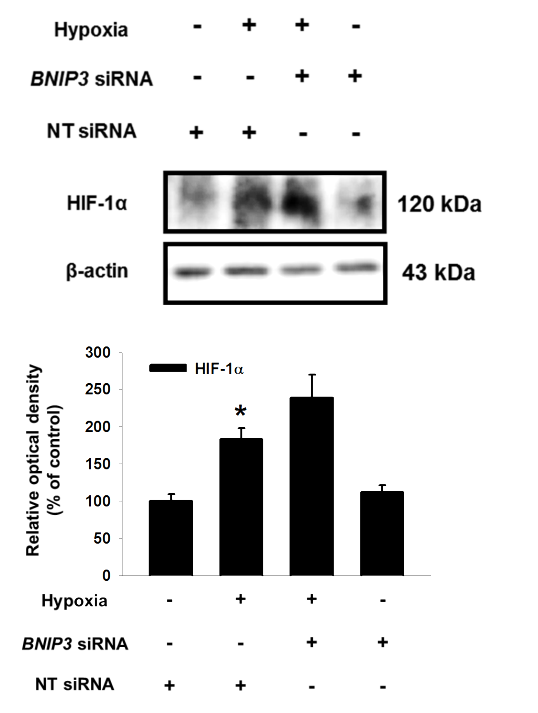


**Supplementary Figure S3. The effect of BNIP3 knock down on HIF-1α expression under hypoxia.** UCB-hMSCs were transfected with *BNIP3* or NT siRNAs prior to hypoxia treatment for 24 h. HIF-1α and β-actin protein expressions were analyzed by western blot. Data are presented as a mean ± S.E.M. *n = 3.* All blot images are representative. . **p* < 0.05 versus control, *#p* < 0.05 versus hypoxia.


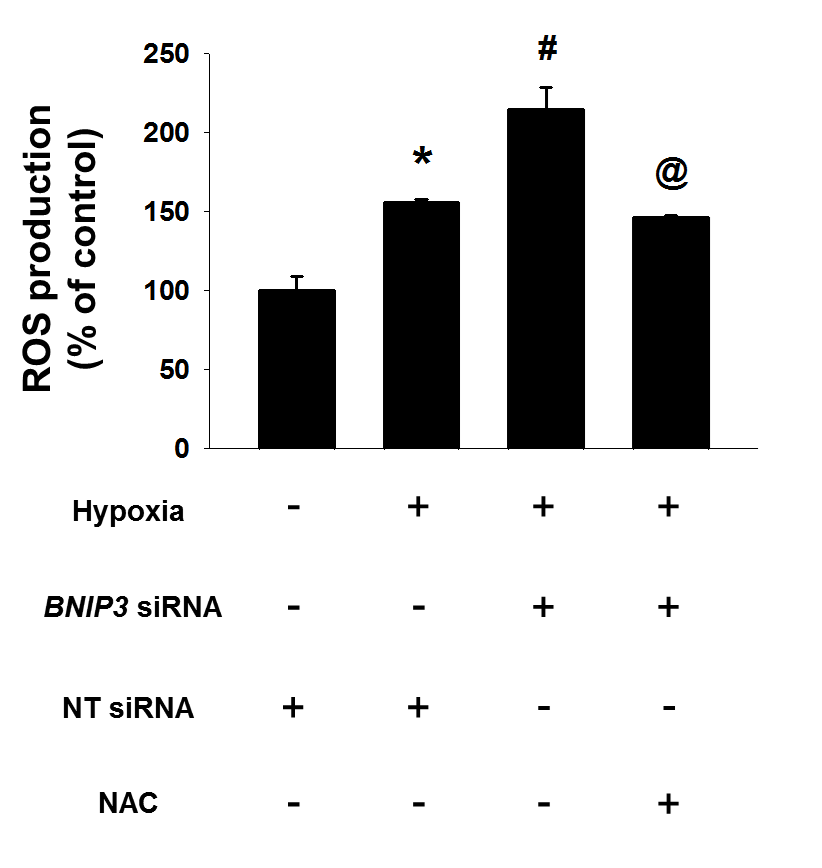


**Supplementary Figure S4. The effect of NAC on ROS induced by BNIP3 knock down in UCB-hMSCs under hypoxia.** UCB-hMSCs were transfected with *BNIP3* or NT siRNAs, and pretreated with NAC (500 uM) prior to hypoxia treatment for 48 h. ROS production level was measured by luminometer described in Materials & Methods. Data are presented as a mean ± S.E.M. *n = 8. *p* < 0.05 versus control, *#p* < 0.05 versus hypoxia, *@p* < 0.05 versus *BNIP3* siRNA-transfected UCB-hMSC with hypoxia.


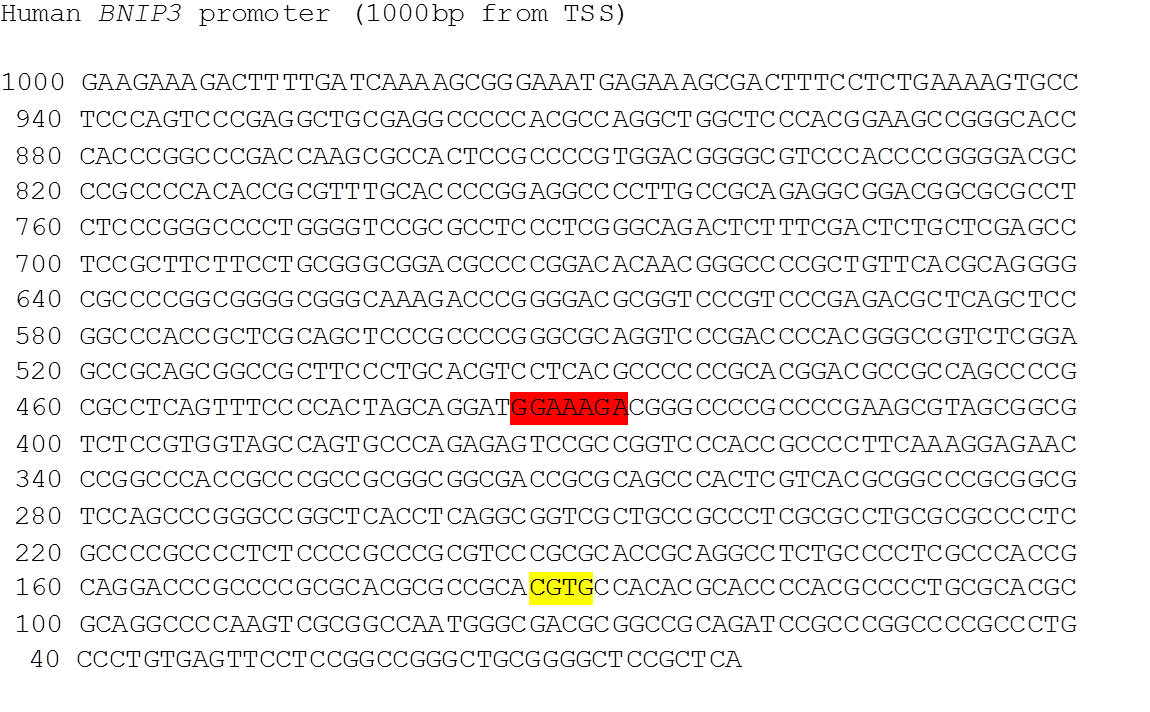


**Supplementary Figure S5. The target sites for HIF-1α and FOXO3 binding to the *BNIP3* gene promoter region.** The CHIP primers were designed referring to the hypoxia responsive element (yellow) and FOXO3 consensus binding sequence (red), respectively.


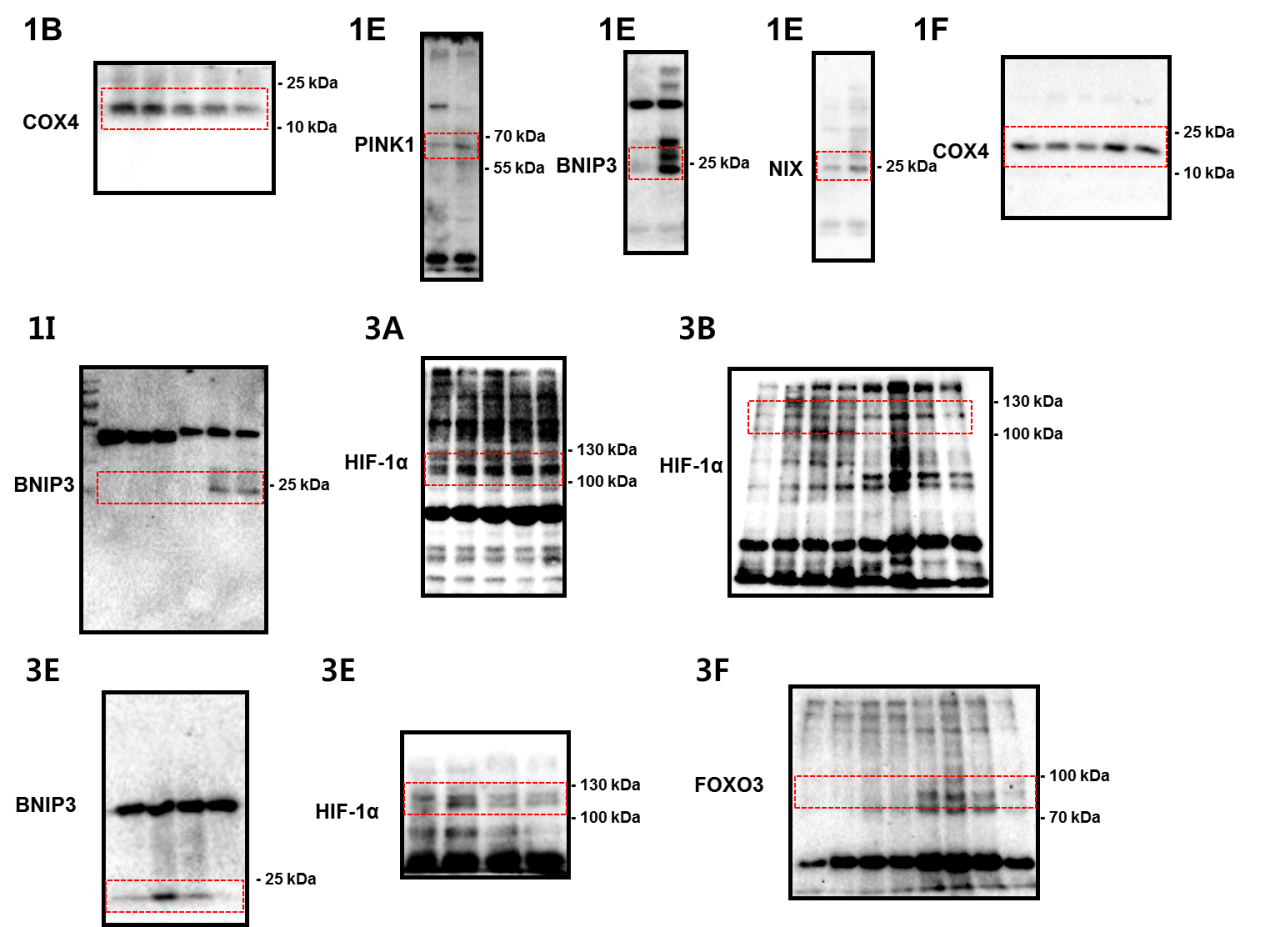


**Supplementary Figure S6. Full-length gel images for key data in figures 1B-3F.** All western blot images are full-length blot images of key blot data in the figures 1B-3F. Dash line box indicates cropped blot image in the figures 1B-3F.


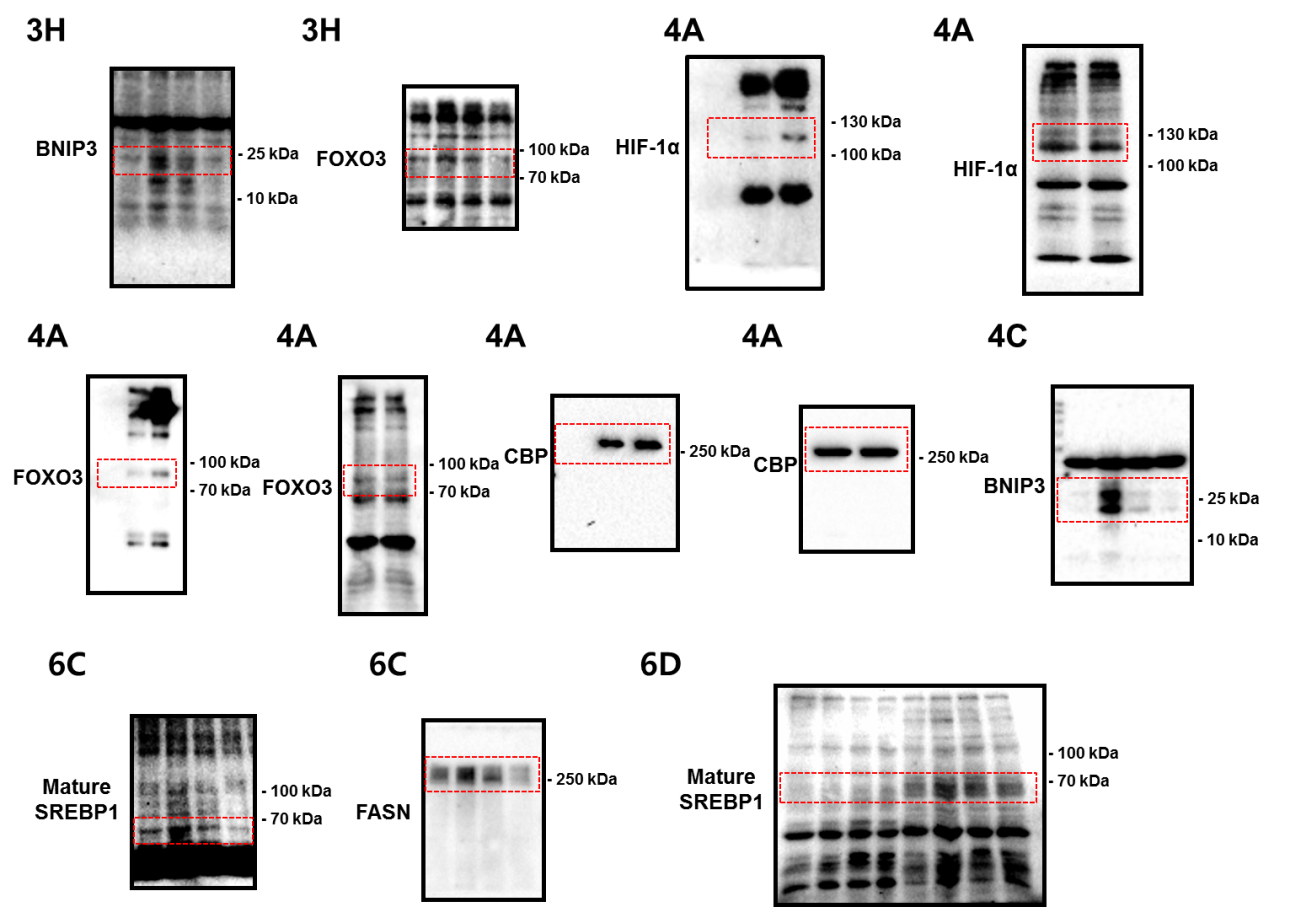


**Supplementary Figure S7. Full-length gel images for key data in figures 3H-6D.** All western blot images are full-length blot images of key blot data in the figures 3H-6D. Dash line box indicates cropped blot image in the figures 3H-6D.


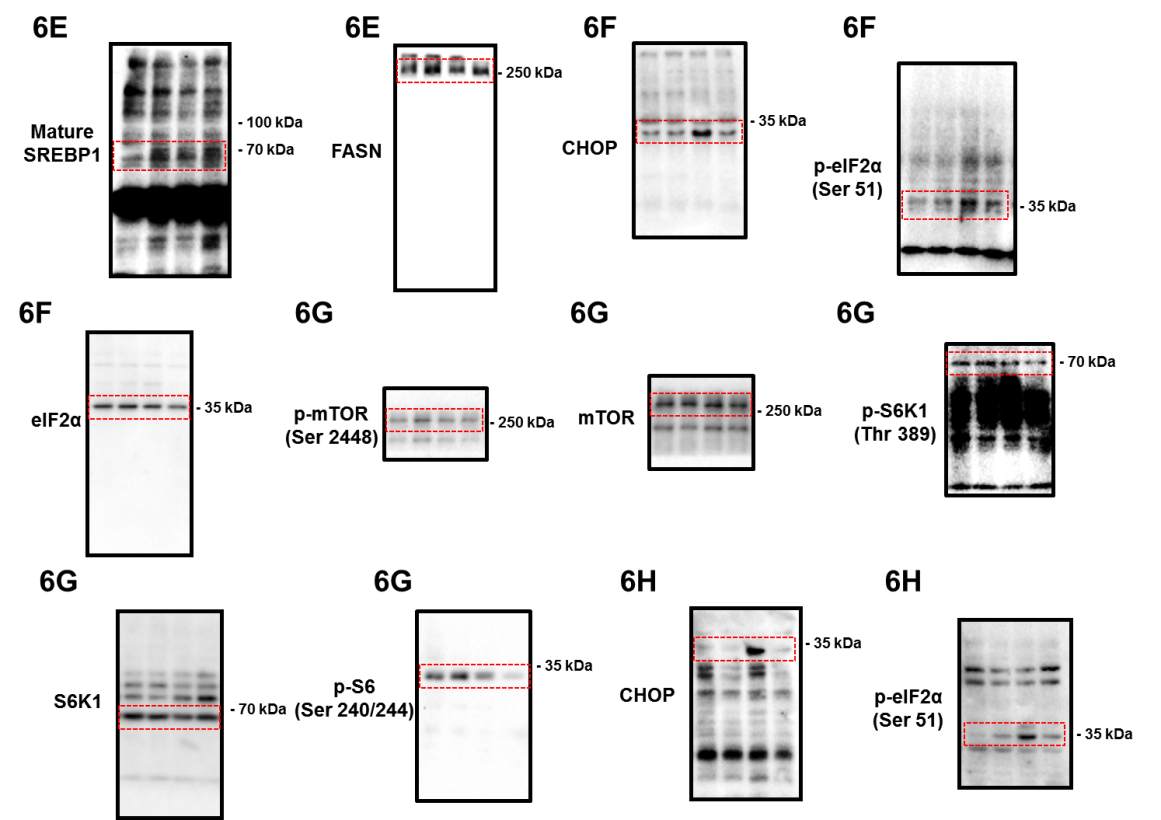


**Supplementary Figure S8. Full-length gel images for key data in figures 6E-6H.** All western blot images are full-length blot images of key blot data in the figures 6E-6H. Dash line box indicates cropped blot image in the figures 6E-6H.


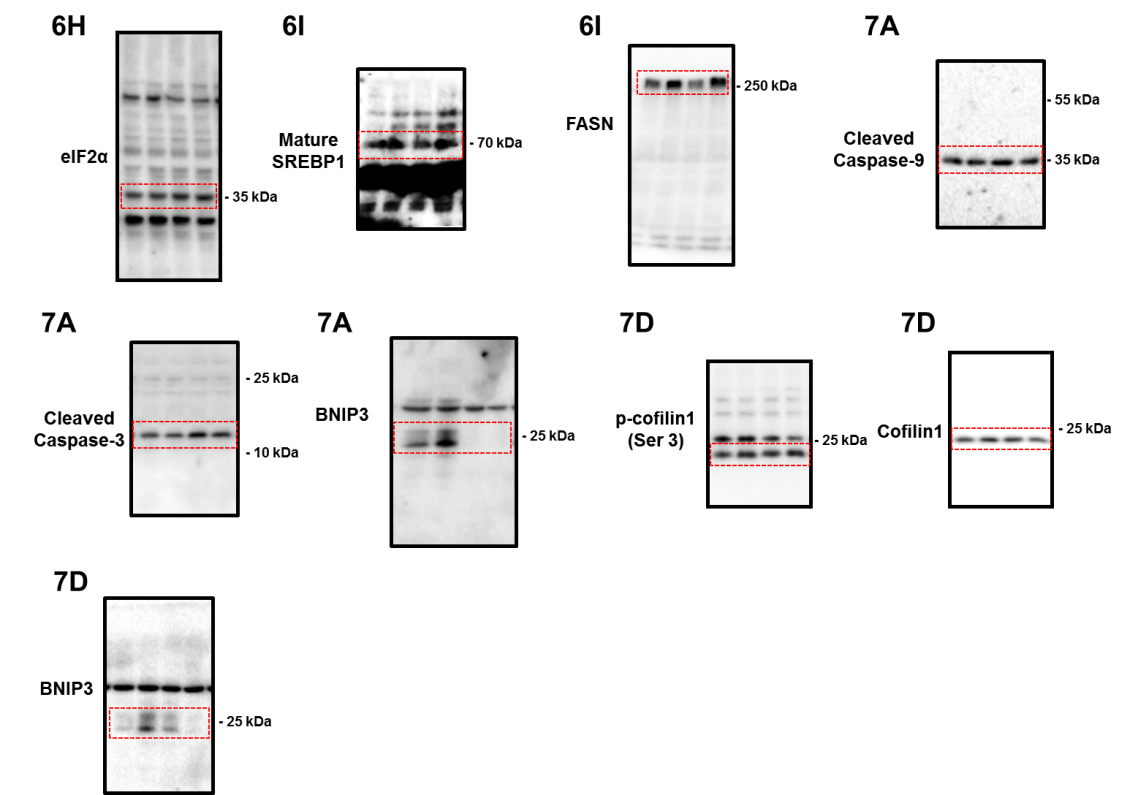


**Supplementary Figure S9. Full-length gel images for key data in figures 6H-7D.** All western blot images are full-length blot images of key blot data in the figures 6H-7D. Dash line box indicates cropped blot image in the figures 6H-7D.

**Supplementary Tables**

**Supplementary Table S1. Scoring of histological changes in skin wound healing**

| **Score** | **Re-epithelialization** |
| --- | --- |
| **0** | Absence of ephithelial proliferation in > 70 % of the tissue |
| **1** | Poor epidermal organization in > 60 % of the tissue |
| **2** | Incomplete epidermal organization in > 40 % of the tissue |
| **3** | Moderate epithelial proliferation in > 60 % of the tissue |
| **4** | Complete epidermal remodeling in > 80 % of the tissue |

**Supplementary Table S2. Sequences of primers used for RT-PCR and real-time PCR**

| Gene | Identification | Sequence (5'-3') | Size (bp) |
| --- | --- | --- | --- |
| *PINK1* | Sense | GCCTCATCGAGGAAAAACAGG | 114 |
|  | Antisense | GTCTCGTGTCCAACGGGTC |  |
| *BNIP3* | Sense | GCCATCGGATTGGGGATCTAT | 150 |
|  | Antisense | GCCACCCCAGGATCTAACAG |  |
| *NIX* | Sense | GGACTCGGCTTGTTGTGTTG | 194 |
|  | Antisense | TAGCTCCACCCAGGAACTGT |  |
| *FASN* | Sense | CCGAGACACTCGTGGGCTA | 209 |
|  | Antisense | CTTCAGCAGGACATTGATGCC |  |
| *SCD1* | Sense | TTCGTTGCCACTTTCTTGCG | 218 |
|  | Antisense | AAGTTGATGTGCCAGCGGTA |  |
| *SCD5* | Sense | GACCTGCTTGCTGATCCTGT | 237 |
|  | Antisense | AGGGCTGATGTGCTTGTCAT |  |
| *GPAT1* | Sense | AGGACGCAACGTCGAGAAC | 176 |
|  | Antisense | GCAGTACCTCCATCATCCCAAG |  |
| *GPAT3* | Sense | GTACATGCCTCCCATGACTAG | 195 |
|  | Antisense | GATCCGTTGCCCACGATCATC |  |
| *GPAT4* | Sense | TCTGGAACAGCAGCAAATAC | 115 |
|  | Antisense | TCTTCATCTGCCTCTCTAGT |  |
| *MAGL* | Sense | TCGTCAGGGATGTGTTGCAG | 155 |
|  | Antisense | AGGCGAAATGAGTACCATGCC |  |
| *DGAT1* | Sense | GGCTTTCACGGGCATGAT | 116 |
|  | Antisense | CTATTGGCTGTCCGATGATGA |  |
| *CPT1A* | Sense | ATCAATCGGACTCTGGAAACGG | 121 |
|  | Antisense | TCAGGGAGTAGCGCATGGT |  |
| *SREBF1* | Sense | GTGGCGGCTGCATTGAGAGTGAAG | 362 |
|  | Antisense | AGGTACCCGAGGGCATCCGAGAAT |  |
| *ACTB* | Sense | AACCGCGAGAAGATGACC | 351 |
|  | Antisense | AGCAGCCGTGGCCATCTC |  |

**Suppplementary Table S3. Sequences of siRNAs used for gene silencing**

| Target gene | Sequence 5'-3' | Supplier |
| --- | --- | --- |
| *PINK1* | GCAAAUGUGCUUCAUCUAA | Dharmacon |
|  | GCUUUCGGCUGGAGGAGUA |  |
|  | GGACGCUGUUCCUCGUUAU |  |
|  | GAGACCAUCUGCCCGAGUA |  |
| *BNIP3* | UCGCAGACACCACAAGAUA | Dharmacon |
|  | GAACUGCACUUCAGCAAUA |  |
|  | GGAAAGAAGUUGAAAGCAU |  |
|  | ACACGAGCGUCAUGAAGAA |  |
| *NIX* | GACCAUAGCUCUCAGUCAG | Dharmacon |
|  | CAACAACAACUGCGAGGAA |  |
|  | GAAGGAAGUCGAGGCUUUG |  |
|  | GAGAAUUGUUUCAGAGUUA |  |
| *HIF1A* | GCCGCUCAAUUUAUGAAUATT  UAUUCAUAAAUUGAGCGGCTT | GenePharma |
|  | GCCUCUUUGACAAACUUAATT  UUAAGUUUGUCAAAGAGGCTT |  |
|  | CCACCACUGAUGAAUUAAATT  UUUAAUUCAUCAGUGGUGGTT |  |
|  | GCUGGAGACACAAUCAUAUTT  AUAUGAUUGUGUCUCCAGCTT |  |
| *FOXO3* | GCACAGAGUUGGAUGAAGU | Dharmacon |
|  | GUACUCAACUAGUGCAAAC |  |
|  | CGAAUCAGCUGACGACAGU |  |
|  | UAACUUUGAUUCCCUCAUC |  |
| Non-targeting (NT) | UAGCGACUAAACACAUCAA | Dharmacon |
|  | UAAGGCUAUGAAGAGAUAC |  |
|  | AUGUAUUGGCCUGUAUUAG |  |
|  | AUGAACGUGAAUUGCUCAA |  |

| Gene | Identification | Sequence (5'-3') | Size (bp) |
| --- | --- | --- | --- |
| *BNIP3* promoter for HIF-1α | Sense | CTTCCCTGCACGTCCTCAC | 171 |
|  | Antisense | CCGGGTTCTCCTTTGAAGGG |  |
| *BNIP3* promoter for FOXO3 | Sense | TCCCGAGACGCTCAGCTC | 168 |
|  | Antisense | TCCATCCTGCTAGTGGGGAA |  |

**Supplementary Table S4. Sequences of CHIP primers used for RT-PCR and real-tim PCR**
